# Supplementary figures and images for: CVB3 VP1 interacts with MAT1 to inhibit cell proliferation by interfering with Cdk-activating kinase complex activity in CVB3-induced acute pancreatitis
Source: PLoS Pathog. 2021 Feb 8;17(2):e1008992. doi: 10.1371/journal.ppat.1008992 (PMC7895353; doi:10.1371/journal.ppat.1008992)

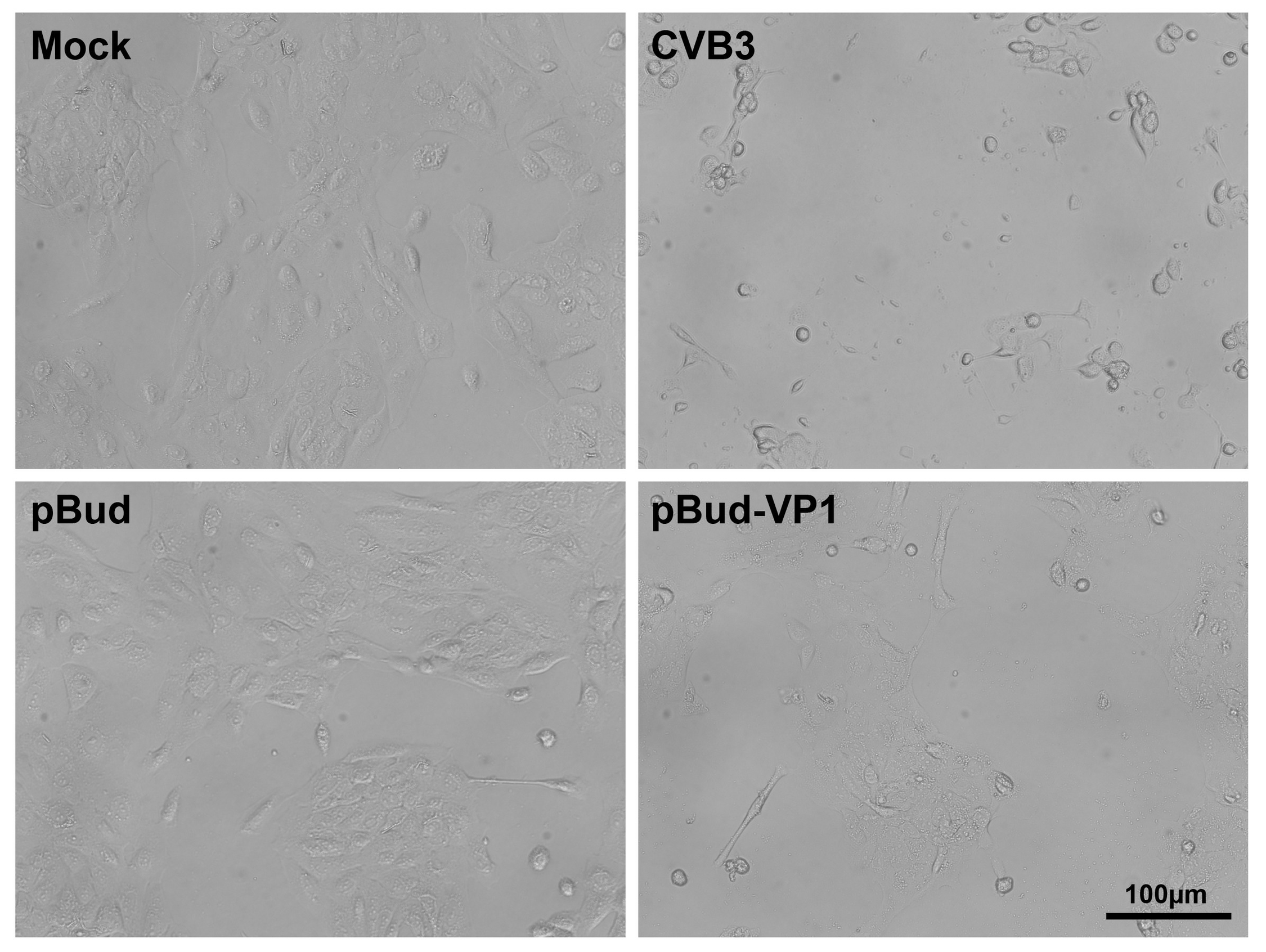

Supplement: S1 Fig — Cell adherence was observed at 48 h after transfection or infection through the comparative analysis of bright-field images. Scale bar = 100 μm. (TIF) [file ppat.1008992.s001.tif]

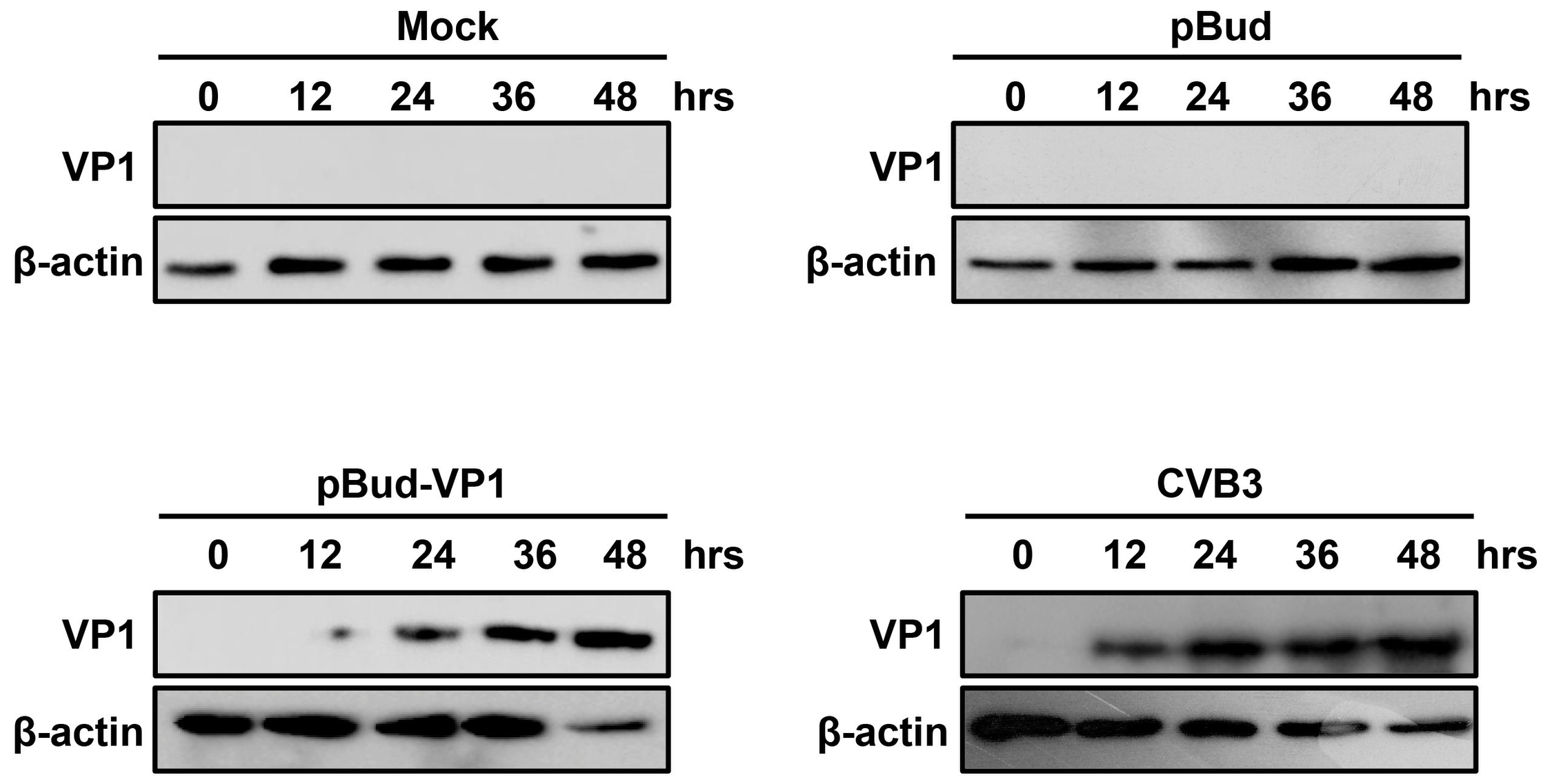

Supplement: S2 Fig — Western blot analysis of VP1 expression in Mock, pBud, pBud-VP1 and CVB3 groups. Original blots are shown in S32 Fig for statistical analysis. (TIF) [file ppat.1008992.s002.tif]

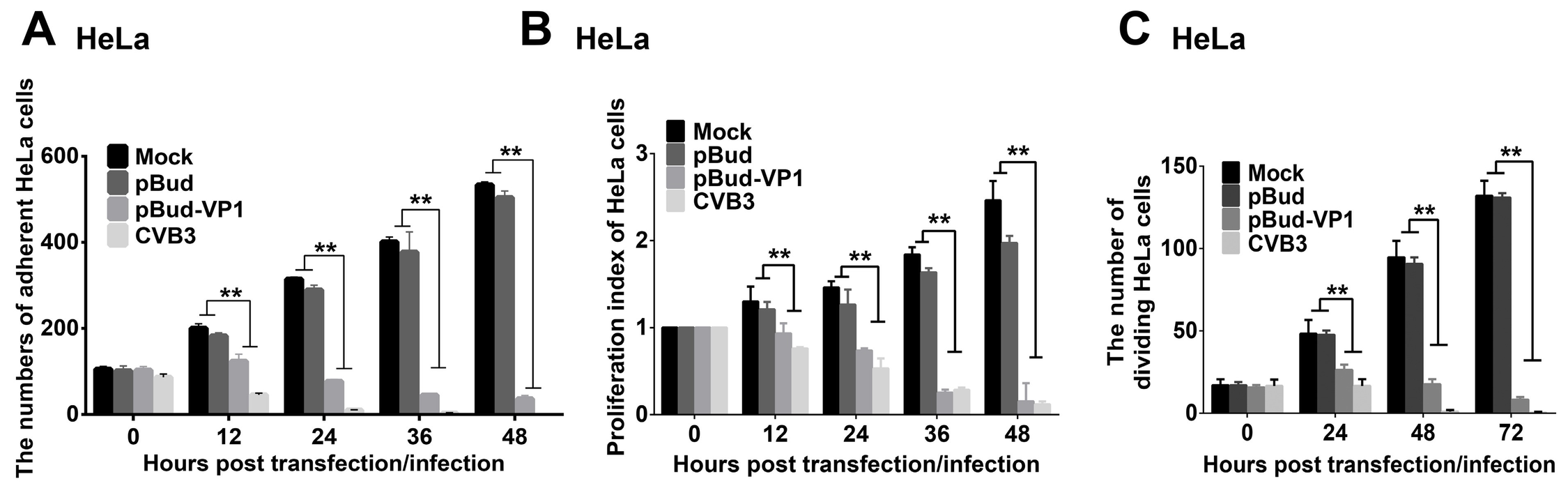

Supplement: S3 Fig — (A) HeLa cells were transfected with pBud-VP1 and pBud plasmid, and infected with Mock and CVB3, and the cell proliferations were separately evaluated using a CCK-8 assay. (B) The activity of DNA replication was examined using EdU incorporation in HeLa cells. (C) The number of adherent HeLa cells in different groups was counted using Image Pro Plus (IPP) software. Mean ± range of values for the counts of cell adherence in replicate experiments. (TIF) [file ppat.1008992.s003.tif]

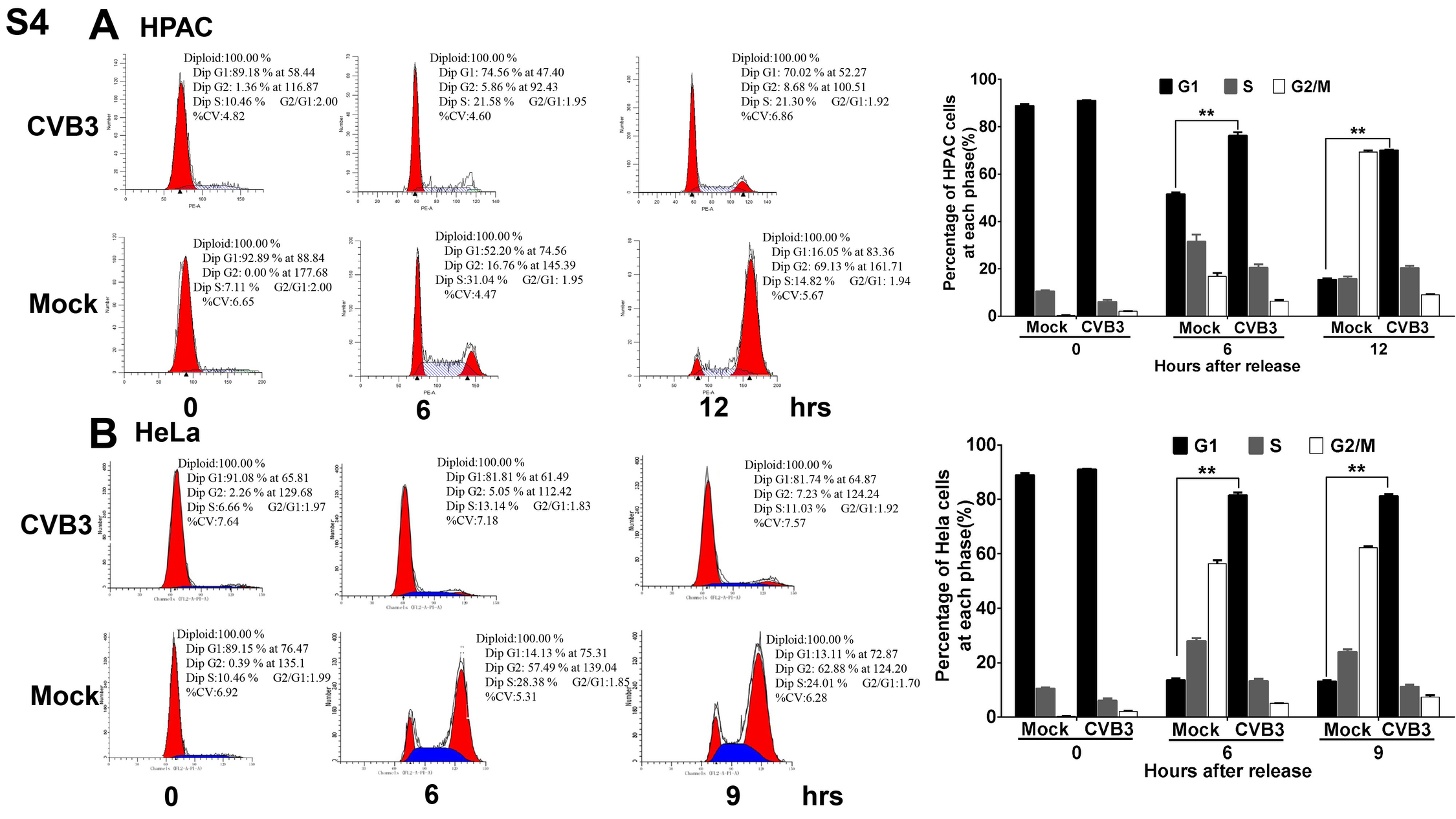

Supplement: S4 Fig — After HPAC and HeLa cells were treated with thymidine again, HPAC (A) and HeLa (B) cells were mock infected or infected with CVB3 at a MOI of 5. These cells were released from the thymidine block and collected according to the indicated release time (0, 6 and 9 or 12 hrs); the cells were analyzed by flow cytometry. The percentage of cells in each phase of the cell cycle is showed as mean ± SEM of three independent experiments. (*P < 0.05, **P < 0.01). (TIF) [file ppat.1008992.s004.tif]

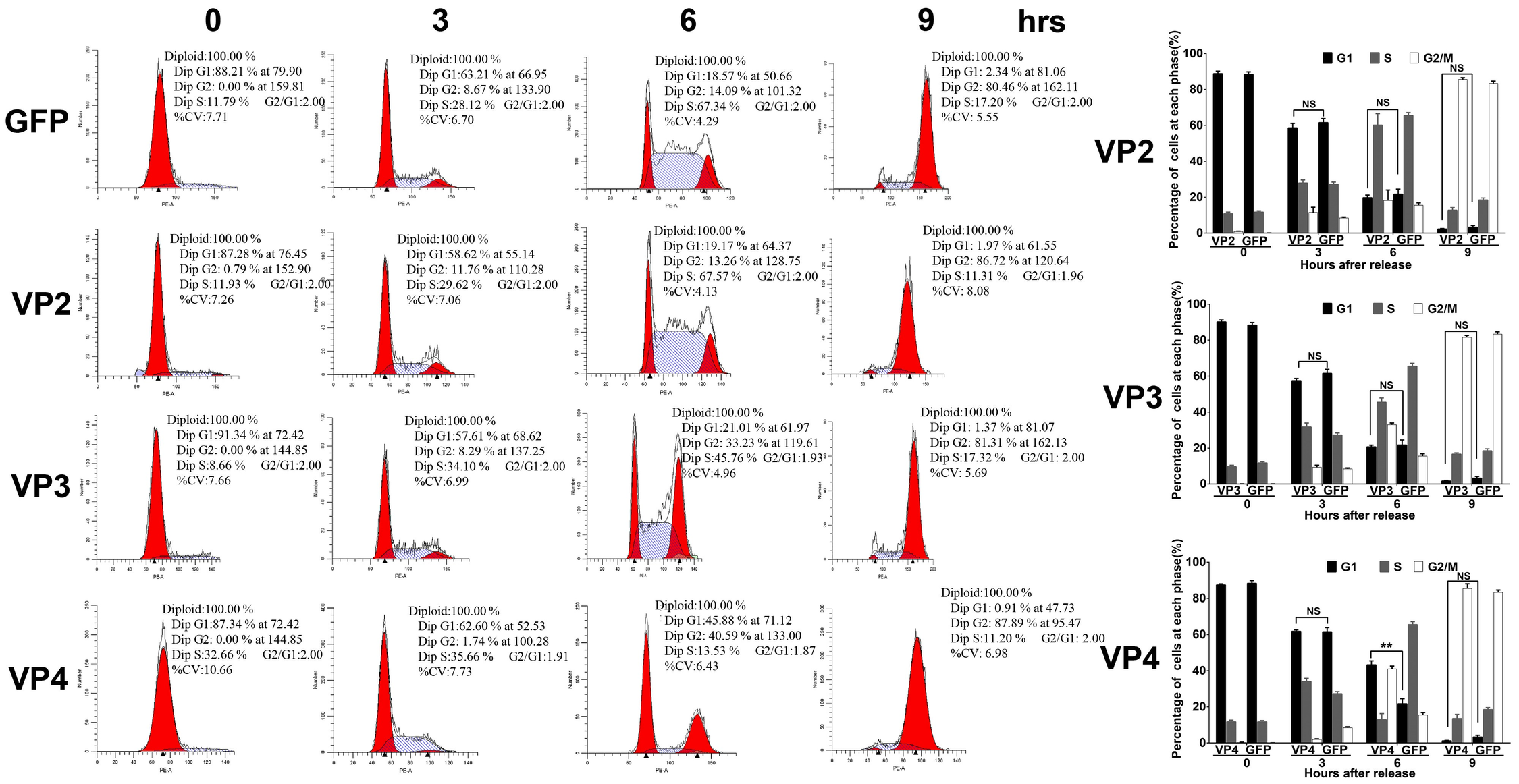

Supplement: S5 Fig — The structural proteins of CVB3 VP2, VP3 and VP4 infected double-thymidine synchronized cells, with GFP as a control. These cells were then released and collected according to the indicated release time (0, 3, 6, or 9 hrs). The percentage of cells in each phase of the cell cycle is shown as mean ± SEM of three independent experiments. (*P < 0.05, **P < 0.01). (TIF) [file ppat.1008992.s005.tif]

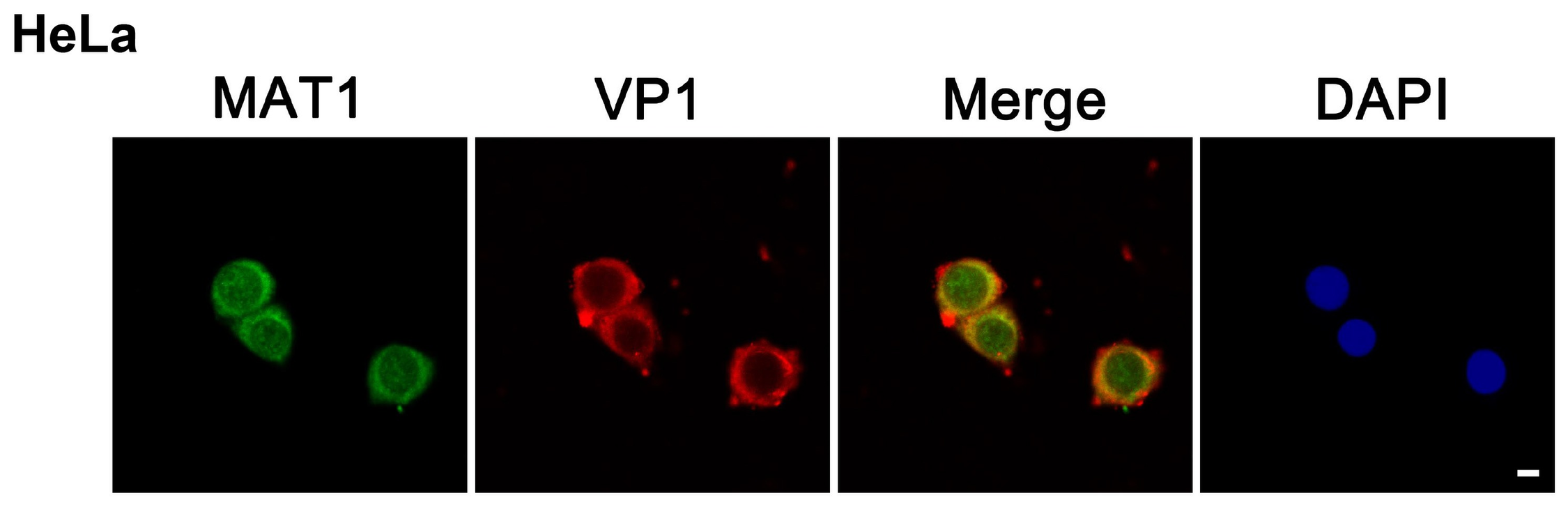

Supplement: S6 Fig — Representative confocal immunofluorescence microscopic images of MAT1 and VP1 stained with rabbit anti-MAT1 (green) and mouse anti-VP1 antibodies (red); the nuclei are labeled with DAPI. Scale bar = 10 μm. (TIF) [file ppat.1008992.s006.tif]

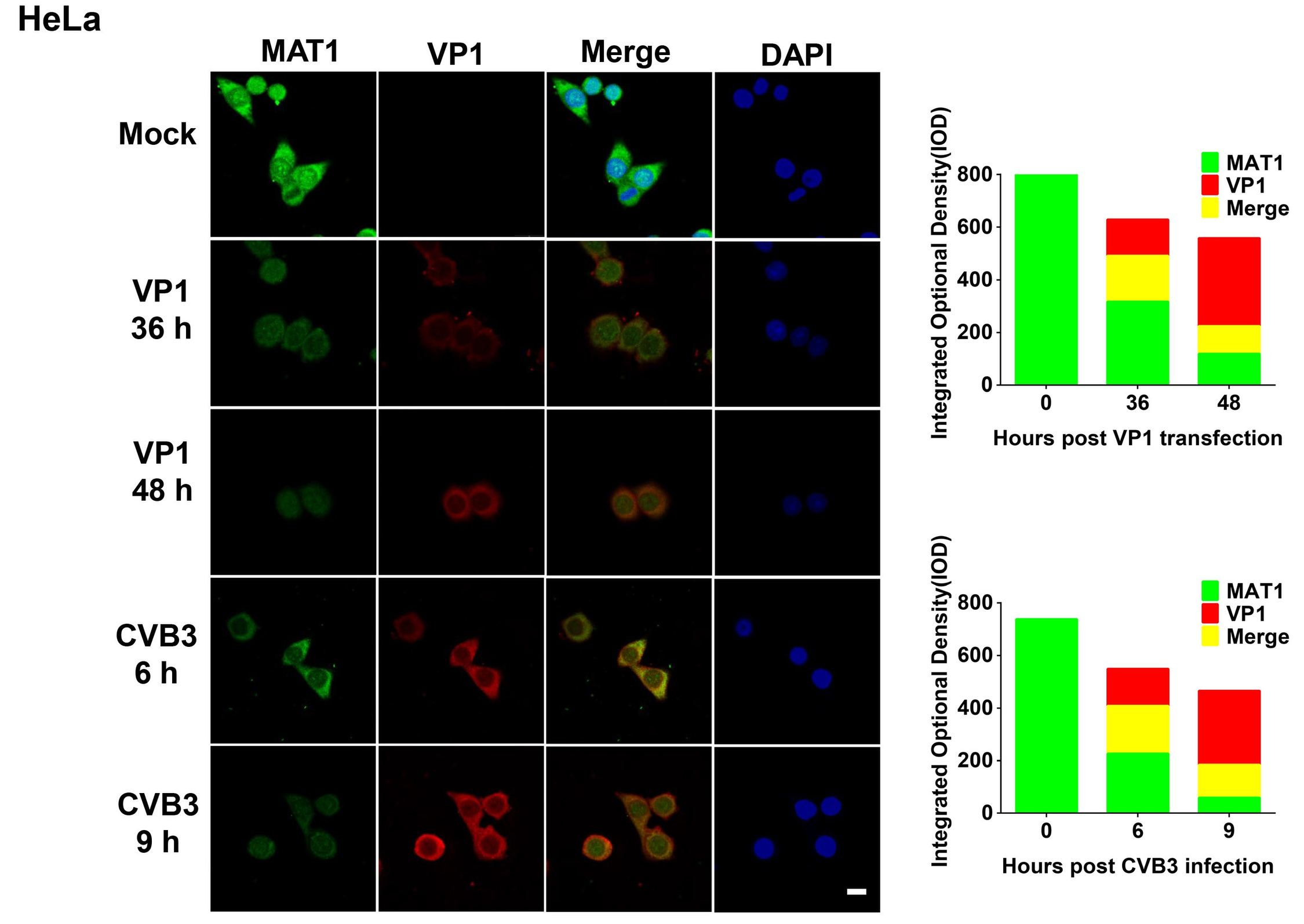

Supplement: S7 Fig — Cells were detected with monoclonal antibodies to MAT1 (Alexa-488) and polyclonal anti-VP1 (fluorescein; red), and counterstained with DAPI to show the nucleus. The MAT1 and VP1 images were merged. Scale bar: 10 μm. (TIF) [file ppat.1008992.s007.tif]

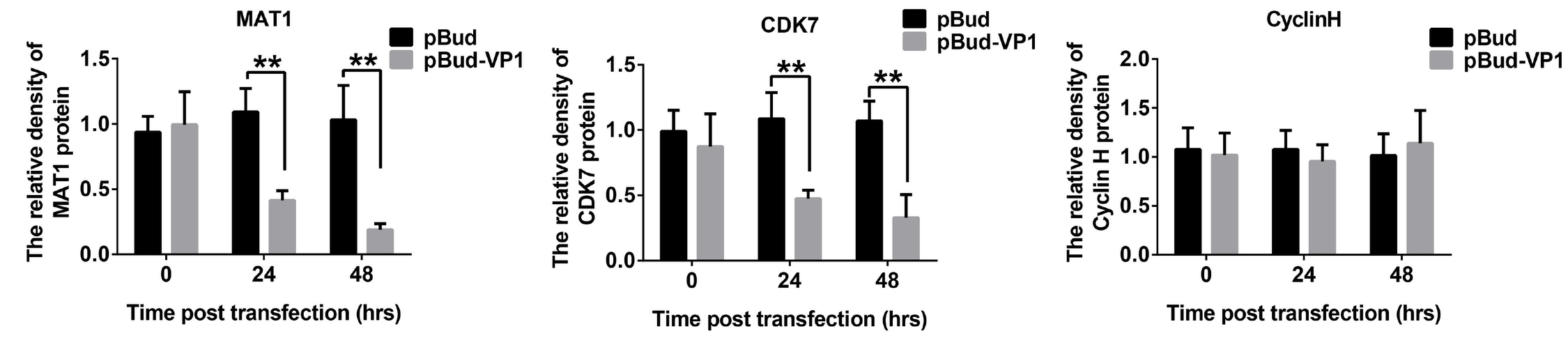

Supplement: S8 Fig — (*P < 0.05, **P < 0.01). (TIF) [file ppat.1008992.s008.tif]

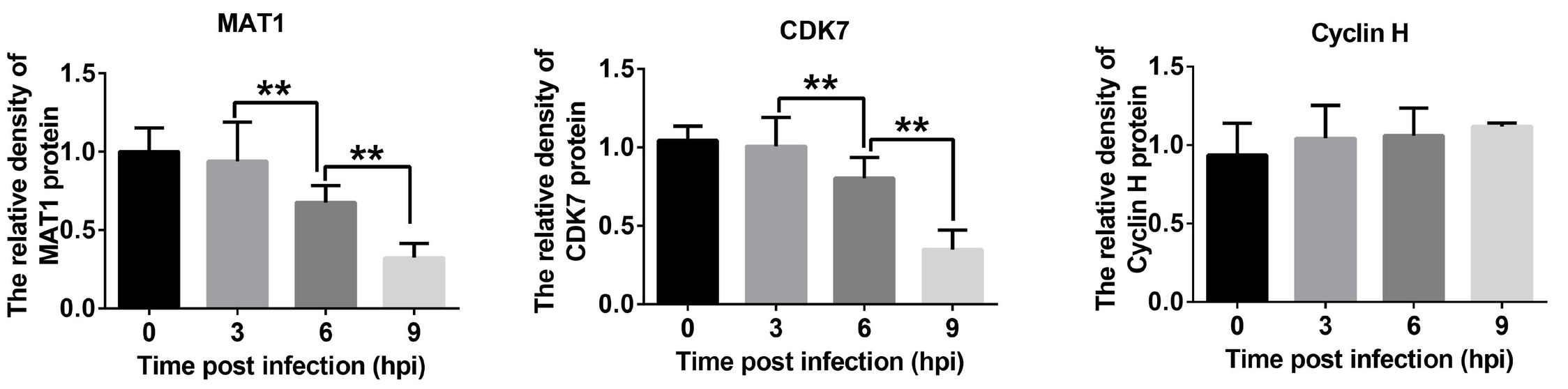

Supplement: S9 Fig — (*P < 0.05, **P < 0.01). (TIF) [file ppat.1008992.s009.tif]

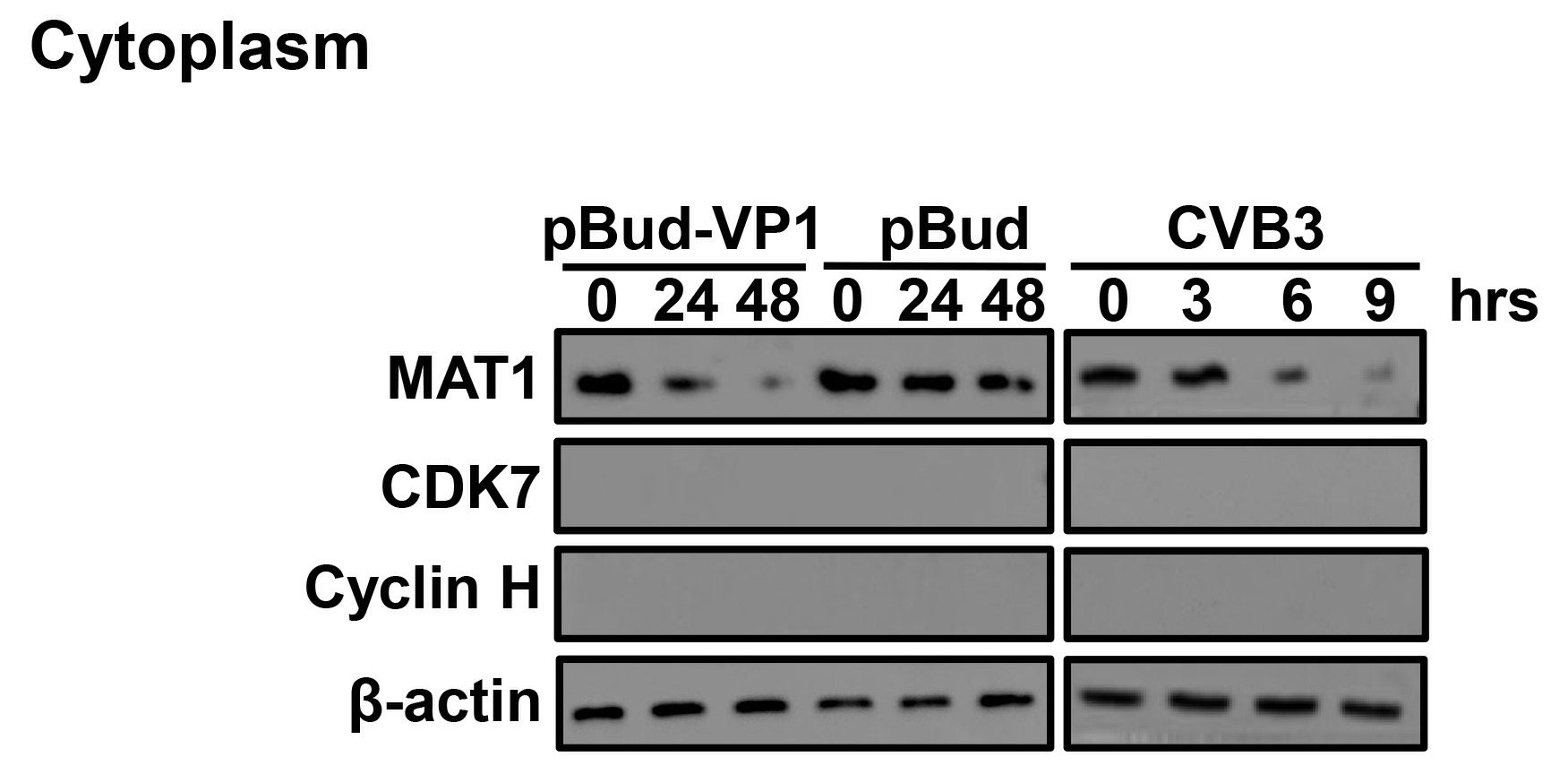

Supplement: S10 Fig — Original blots are shown in S33 Fig for statistical analysis. (TIF) [file ppat.1008992.s010.tif]

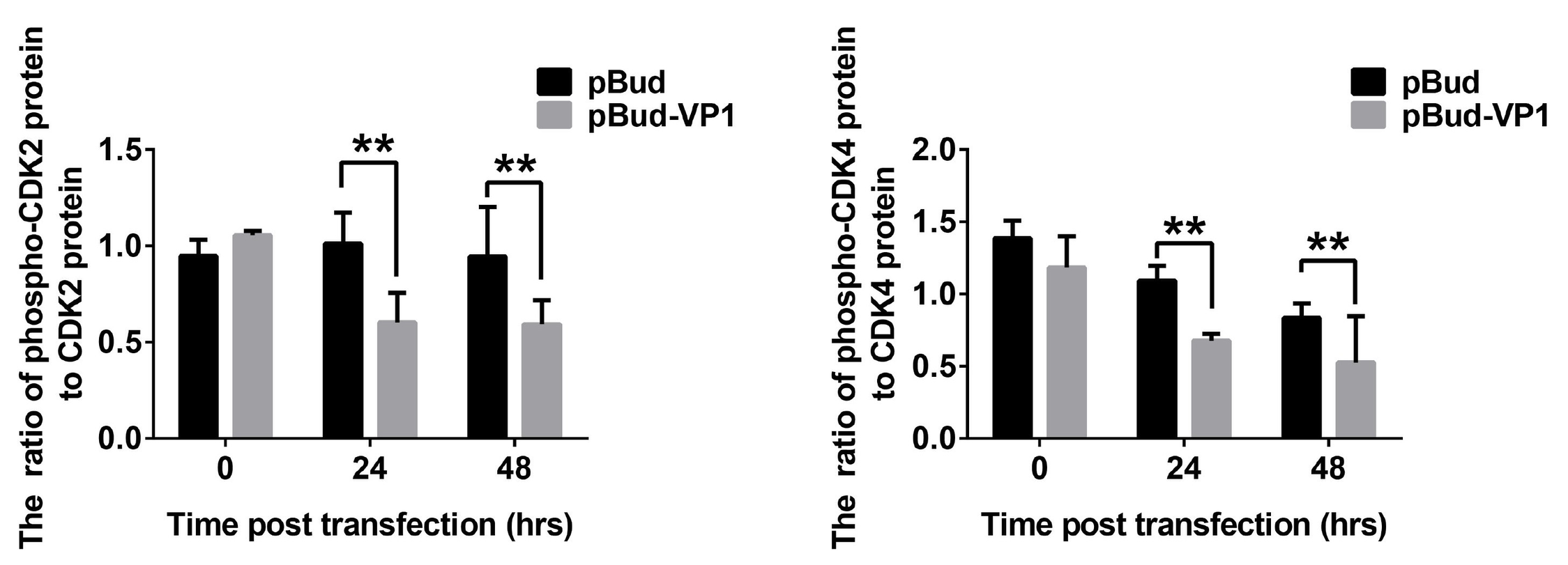

Supplement: S11 Fig — (*P < 0.05, **P < 0.01). (TIF) [file ppat.1008992.s011.tif]

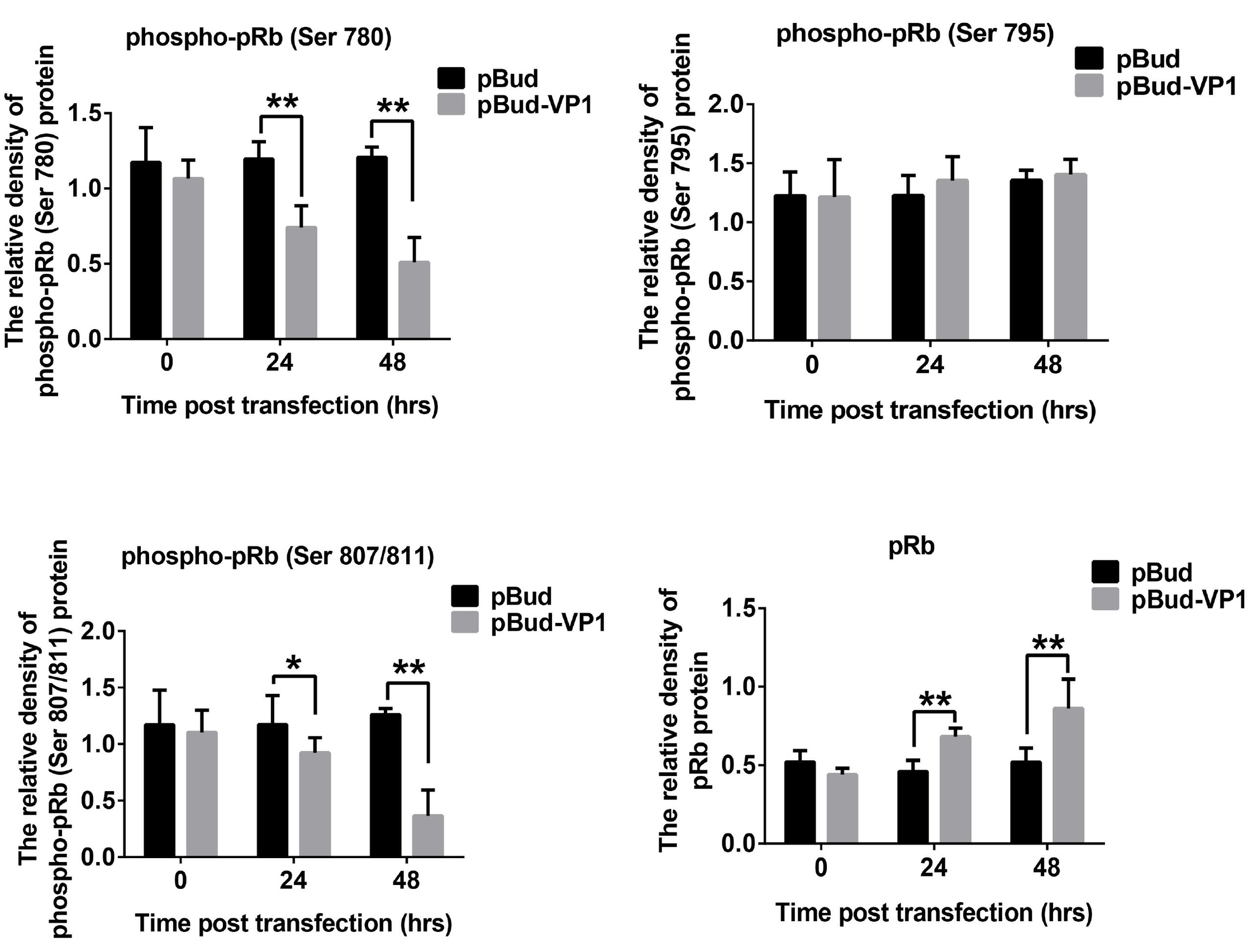

Supplement: S12 Fig — (*P < 0.05, **P < 0.01). (TIF) [file ppat.1008992.s012.tif]

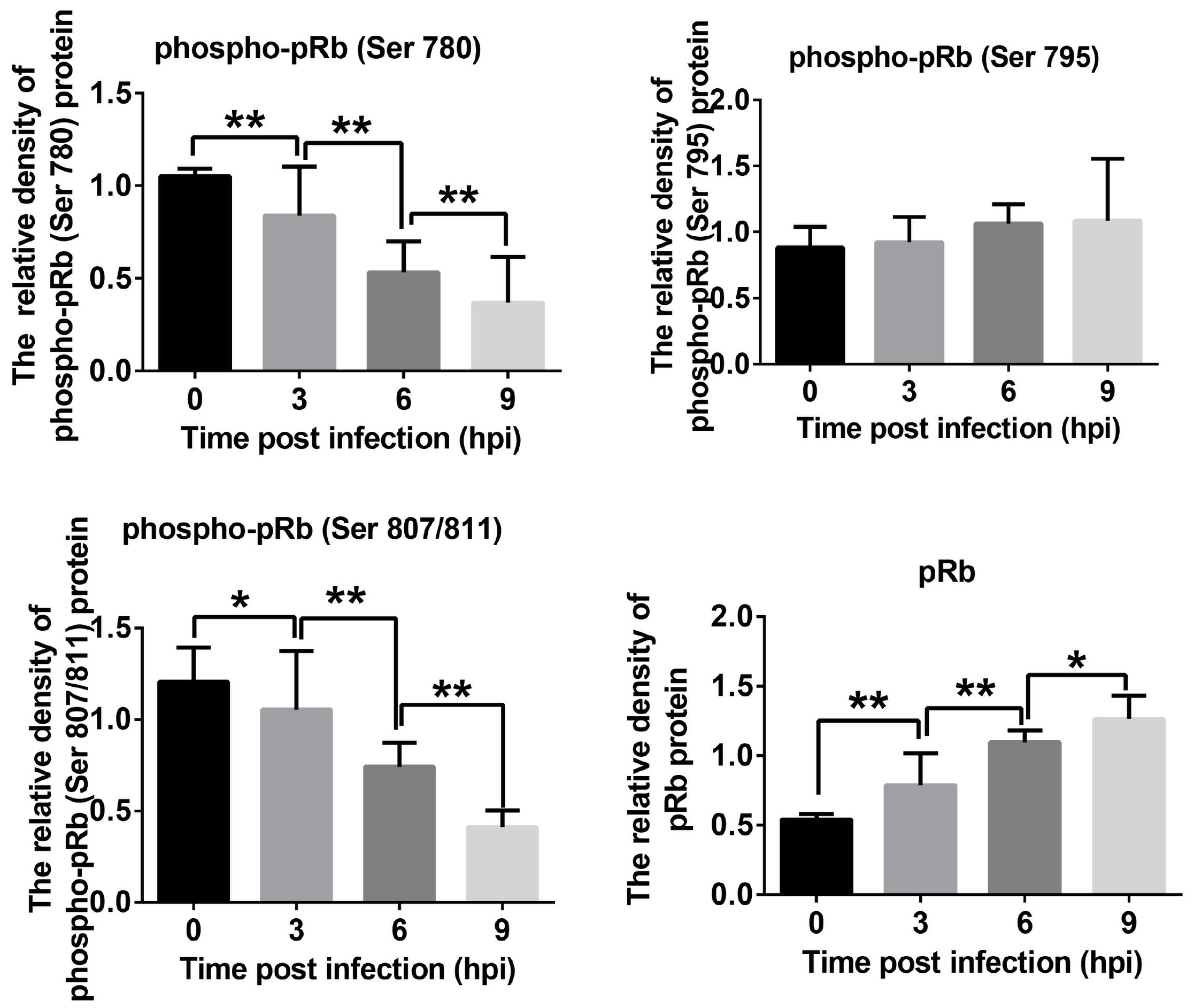

Supplement: S13 Fig — (*P < 0.05, **P < 0.01). (TIF) [file ppat.1008992.s013.tif]

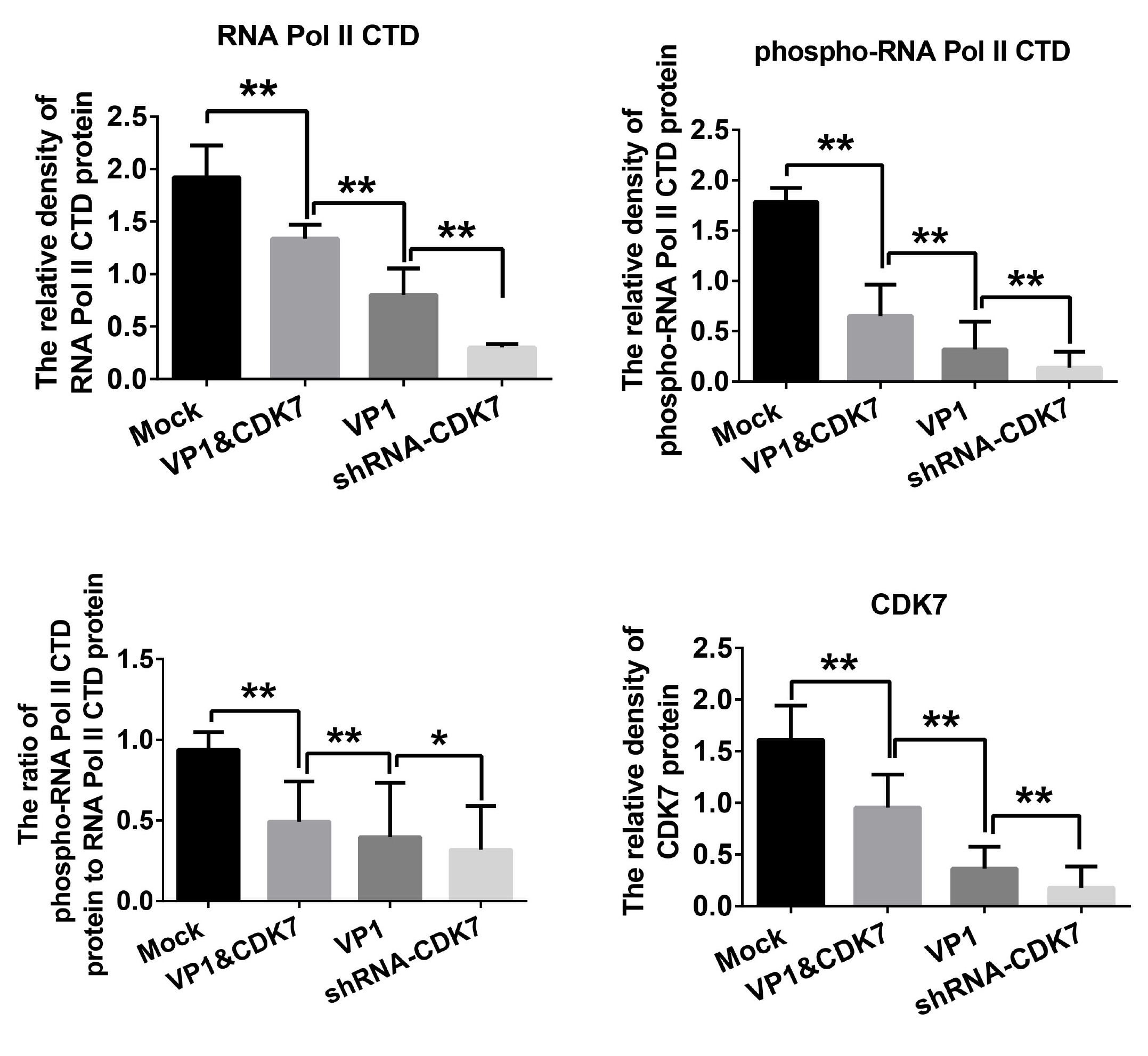

Supplement: S14 Fig — (*P < 0.05, **P < 0.01). (TIF) [file ppat.1008992.s014.tif]

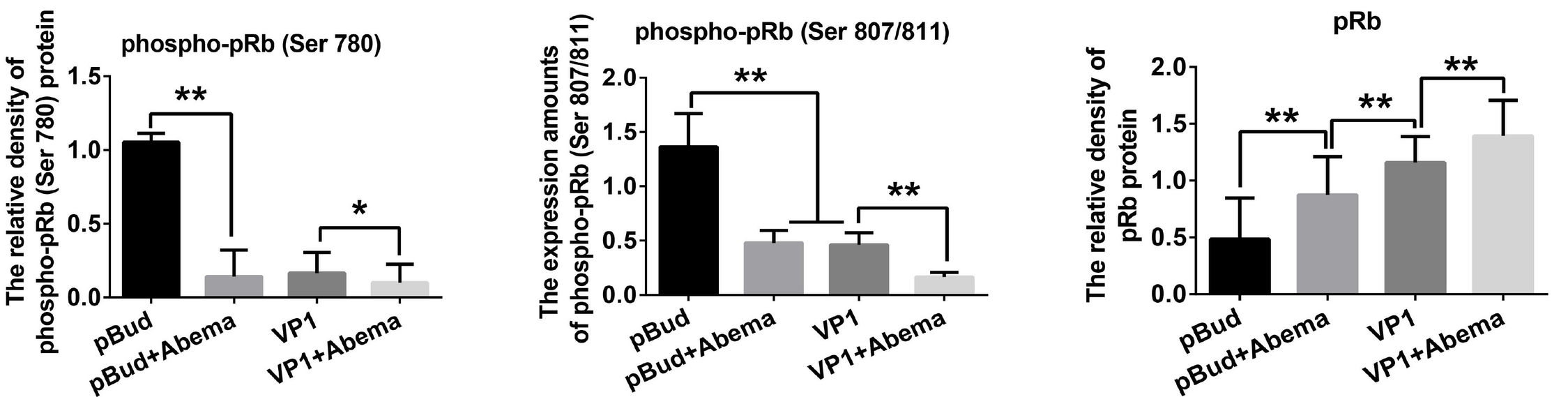

Supplement: S15 Fig — (*P < 0.05, **P < 0.01). (TIF) [file ppat.1008992.s015.tif]

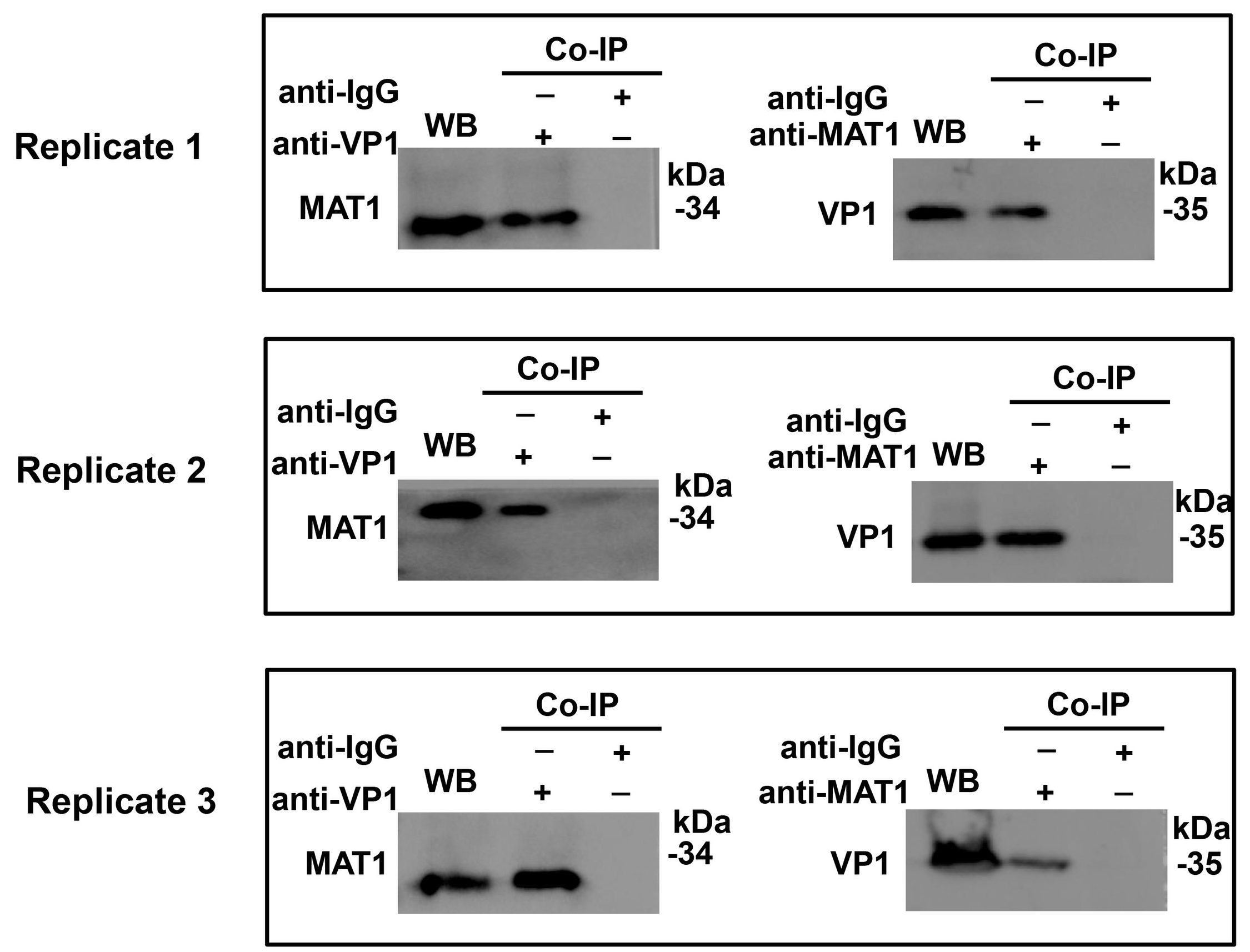

Supplement: S16 Fig — This result shows the raw data of the experiment repeated three times by independent researchers. Related to Fig 2C. (TIF) [file ppat.1008992.s016.tif]

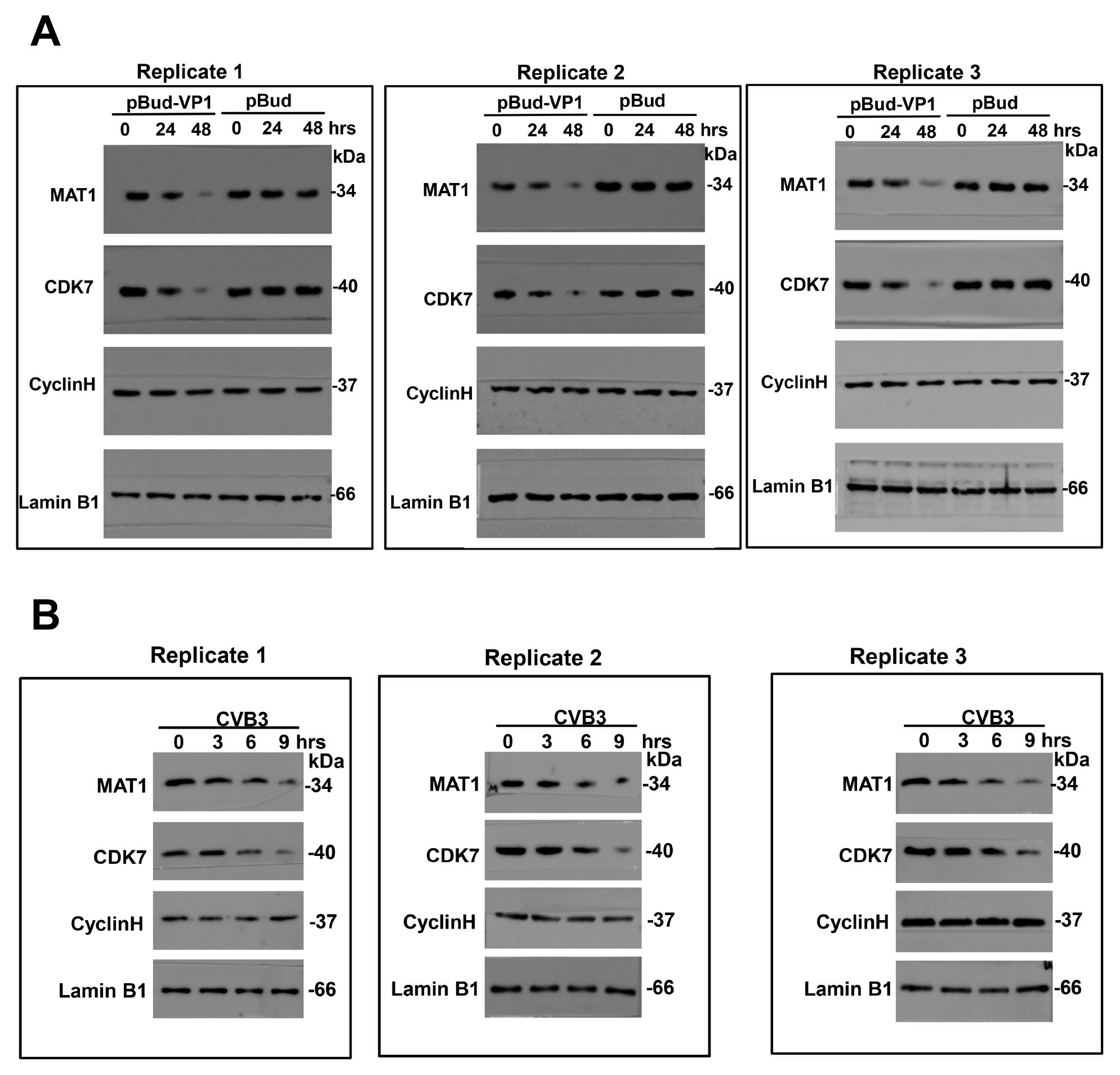

Supplement: S17 Fig — This result shows the raw data of the experiment repeated three times by independent researchers. Related to Fig 3B. (TIF) [file ppat.1008992.s017.tif]

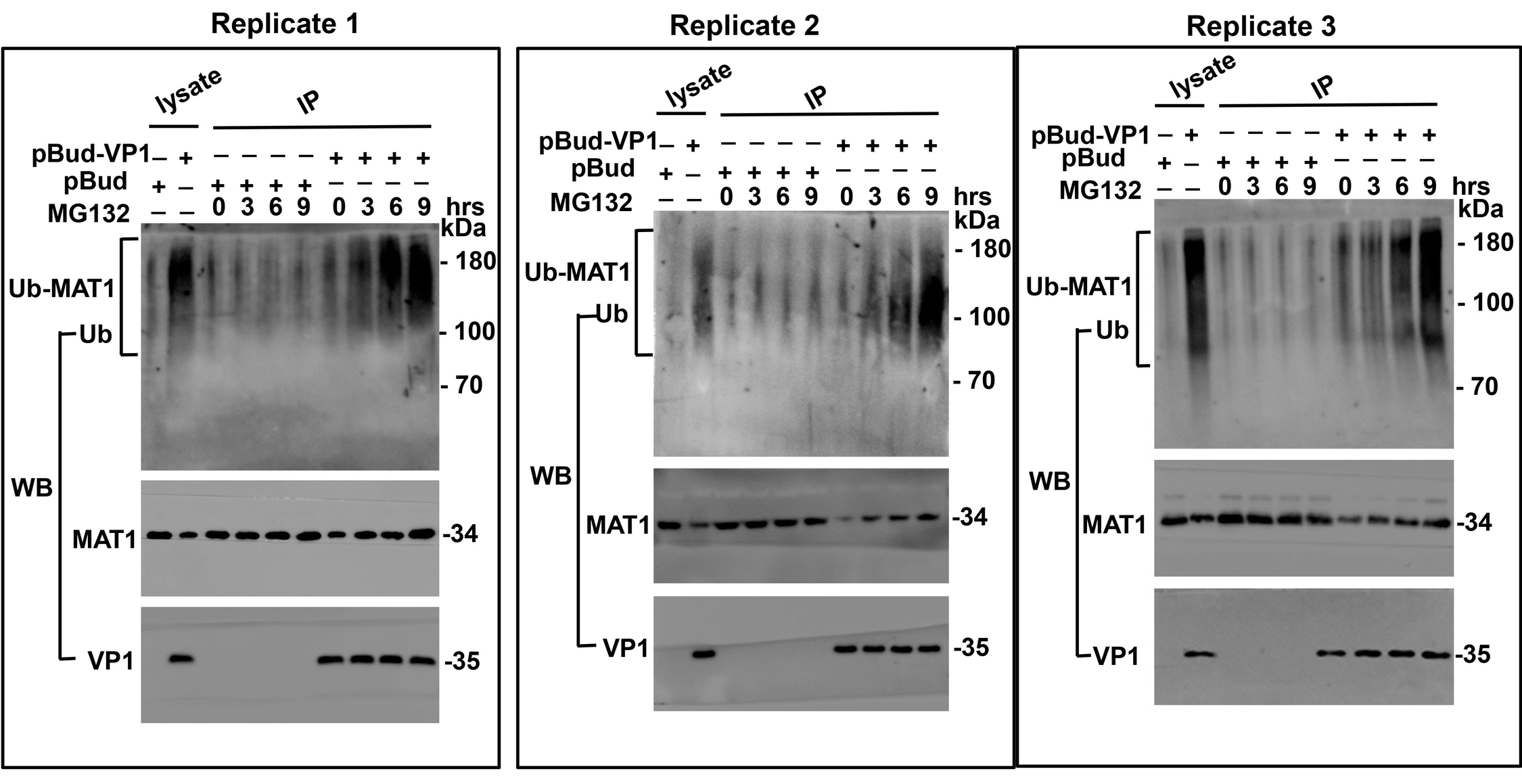

Supplement: S18 Fig — The cell lysates of pBud-VP1 and pBud transfected cells were incubated with monoclonal antibody against MAT1 with 0, 3, 6, 9 hrs. This result shows the raw data of the experiment repeated three times by independent researchers. Related to Fig 3C. (TIF) [file ppat.1008992.s018.tif]

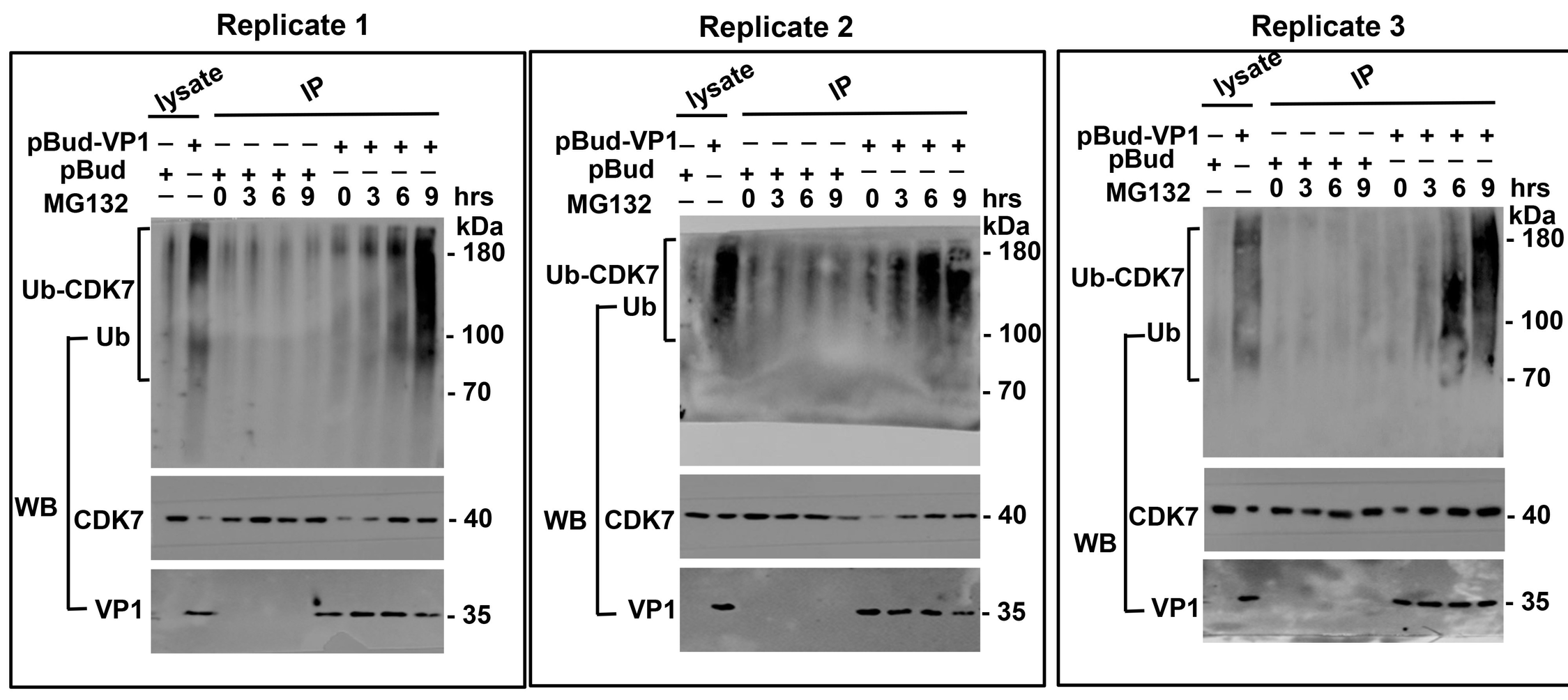

Supplement: S19 Fig — This result shows the raw data of the experiment repeated three times by independent researchers. Related to Fig 3D. (TIF) [file ppat.1008992.s019.tif]

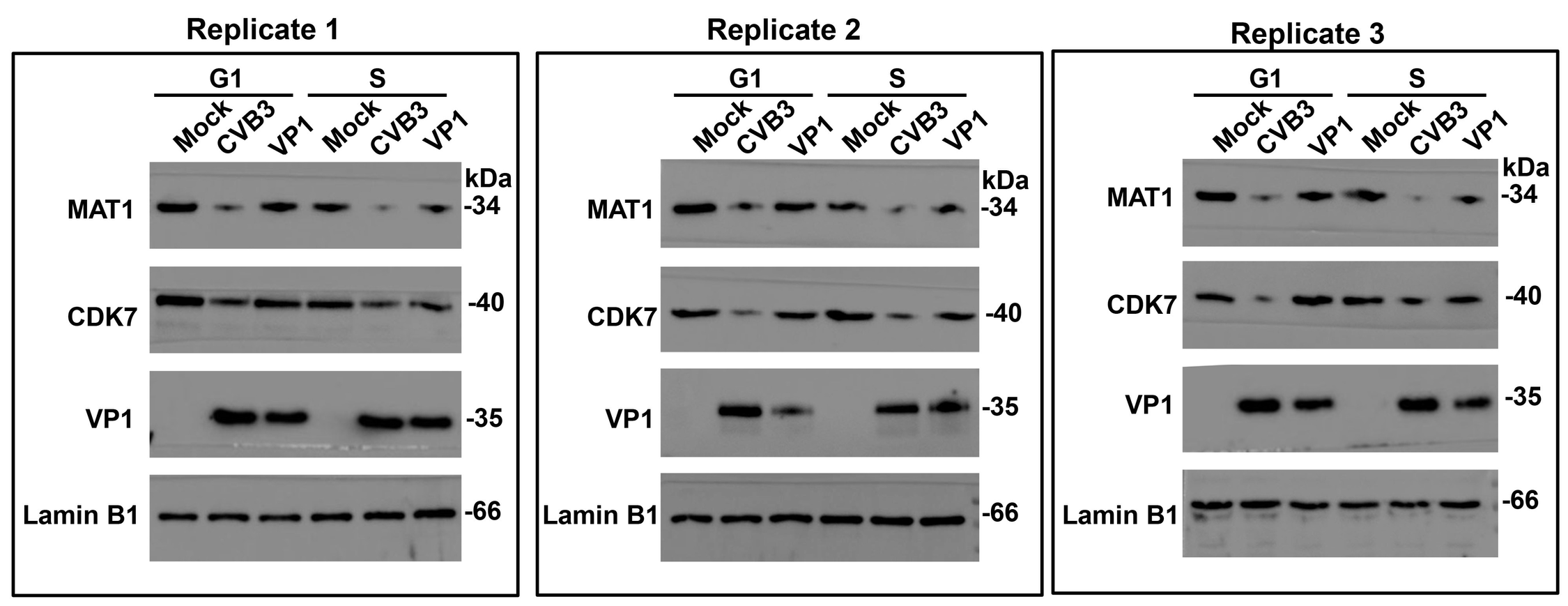

Supplement: S20 Fig — This result shows the raw data of the experiment repeated three times by independent researchers. Related to Fig 4E. (TIF) [file ppat.1008992.s020.tif]

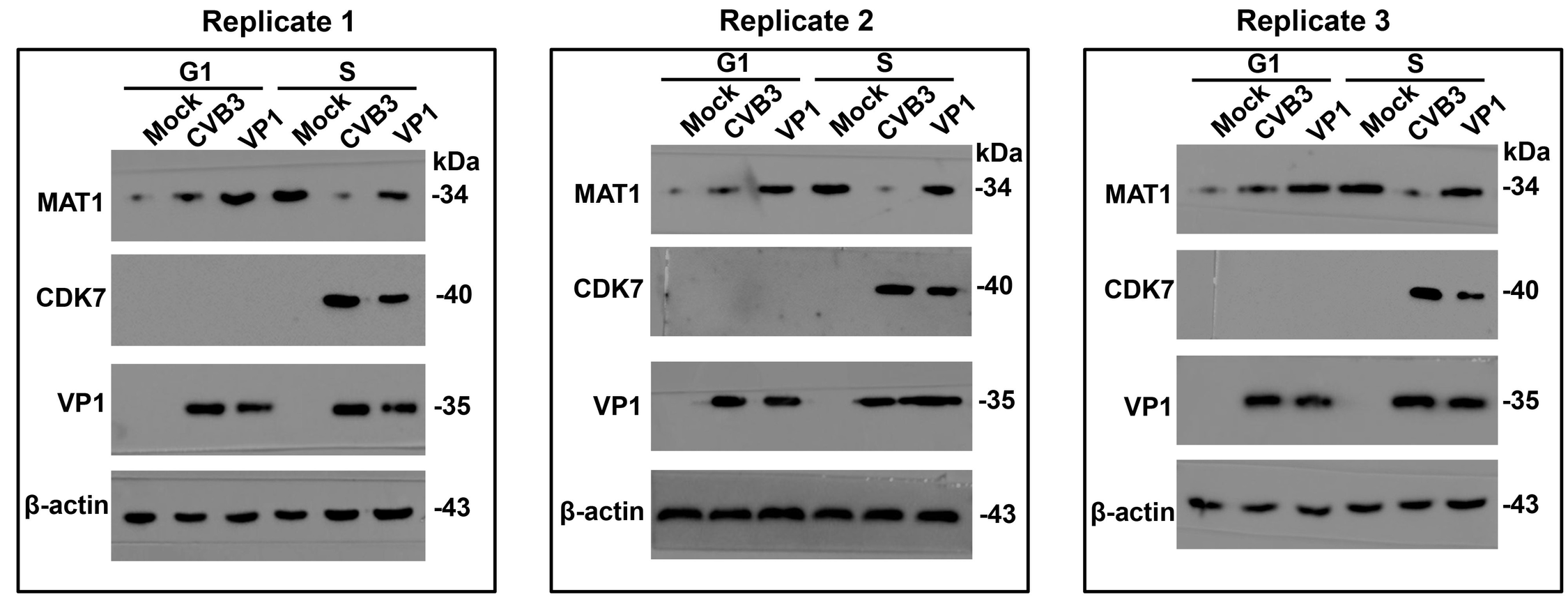

Supplement: S21 Fig — This result shows the raw data of the experiment repeated three times by independent researchers. Related to Fig 4F. (TIF) [file ppat.1008992.s021.tif]

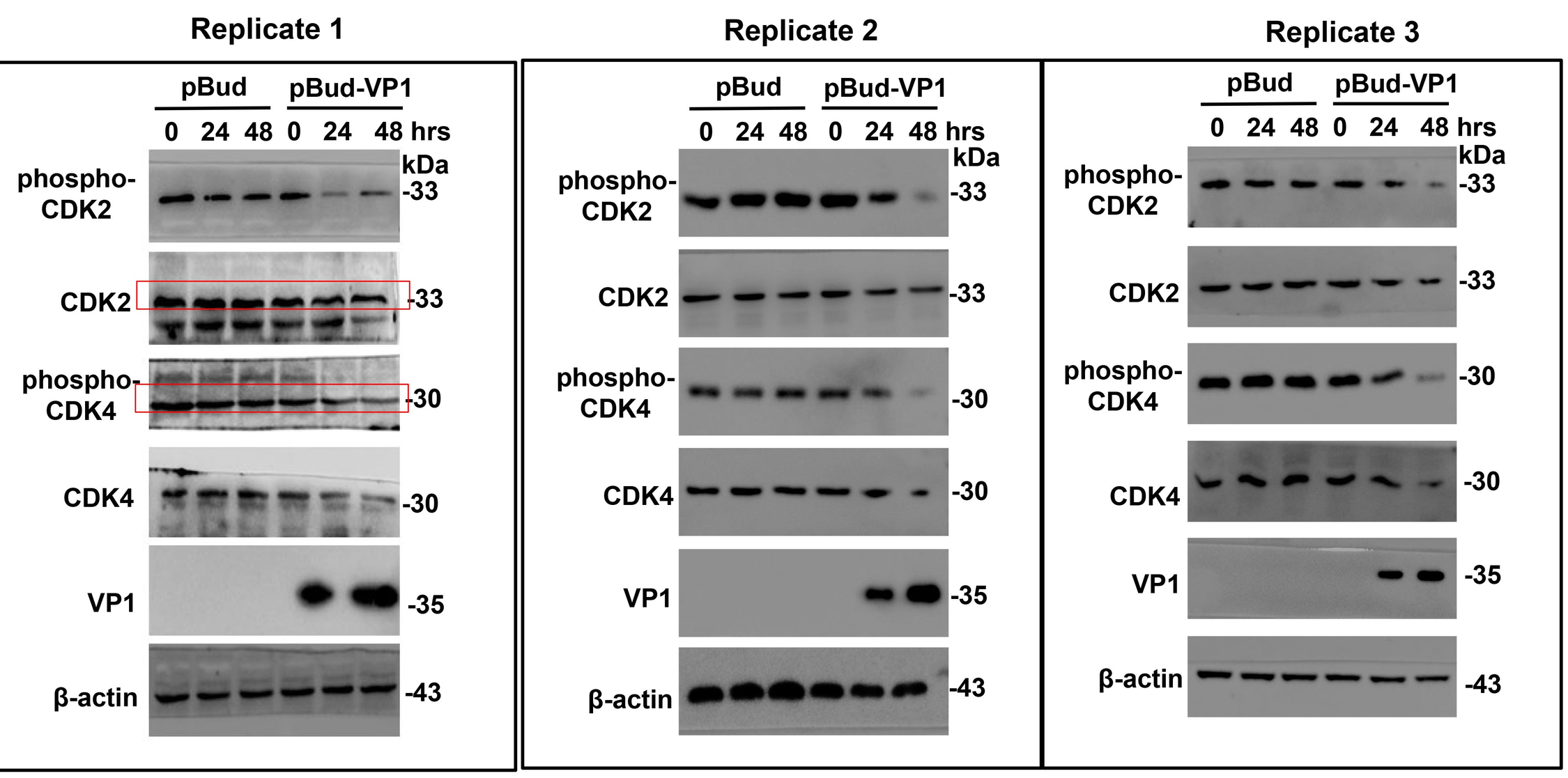

Supplement: S22 Fig — This result shows the raw data of the experiment repeated three times by independent researchers. Related to Fig 5A. (TIF) [file ppat.1008992.s022.tif]

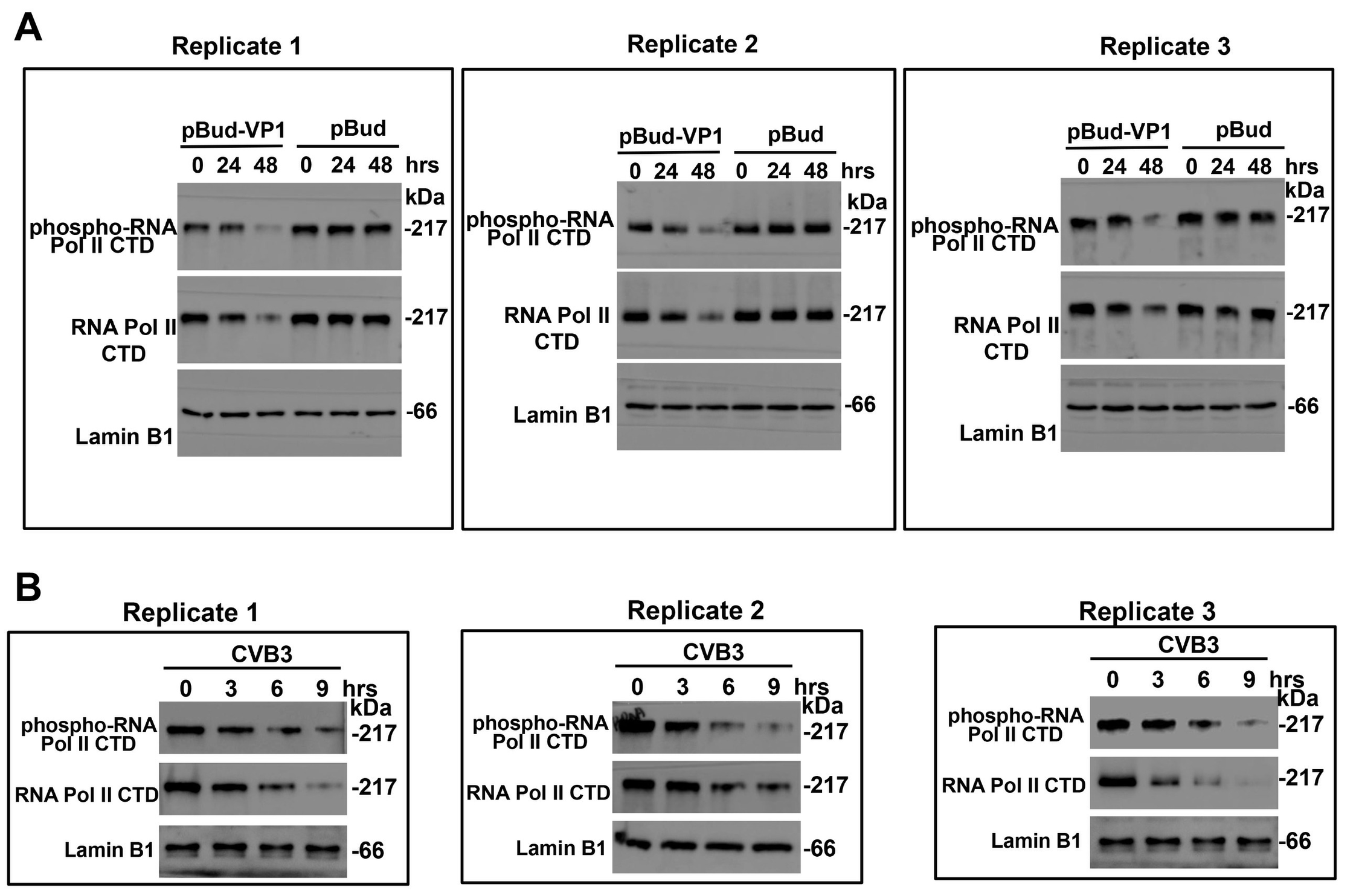

Supplement: S23 Fig — This result shows the raw data of the experiment repeated three times by independent researchers. Related to Fig 5B. (TIF) [file ppat.1008992.s023.tif]

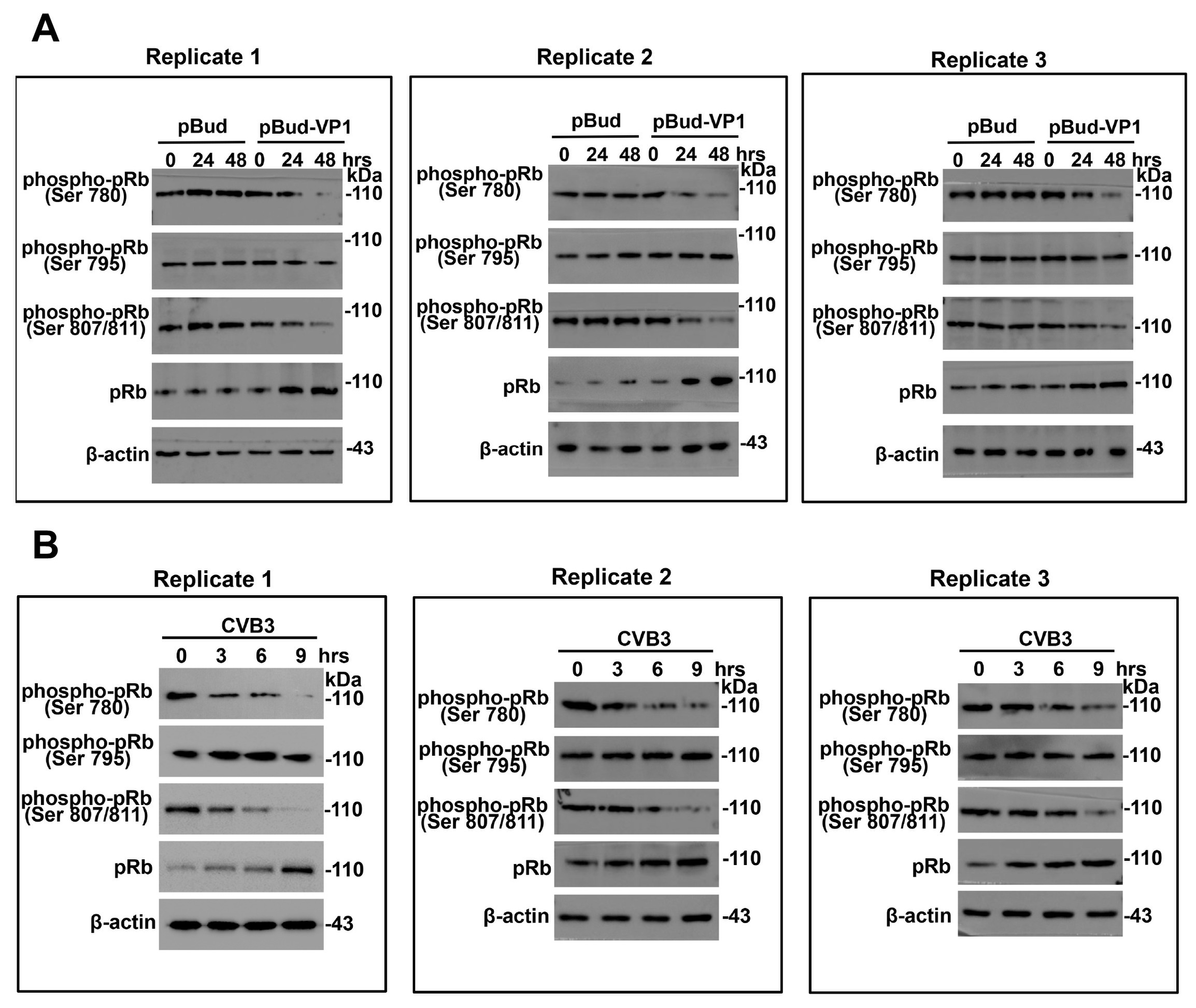

Supplement: S24 Fig — This result shows the raw data of the experiment repeated three times by independent researchers. Related to Fig 5C. (TIF) [file ppat.1008992.s024.tif]

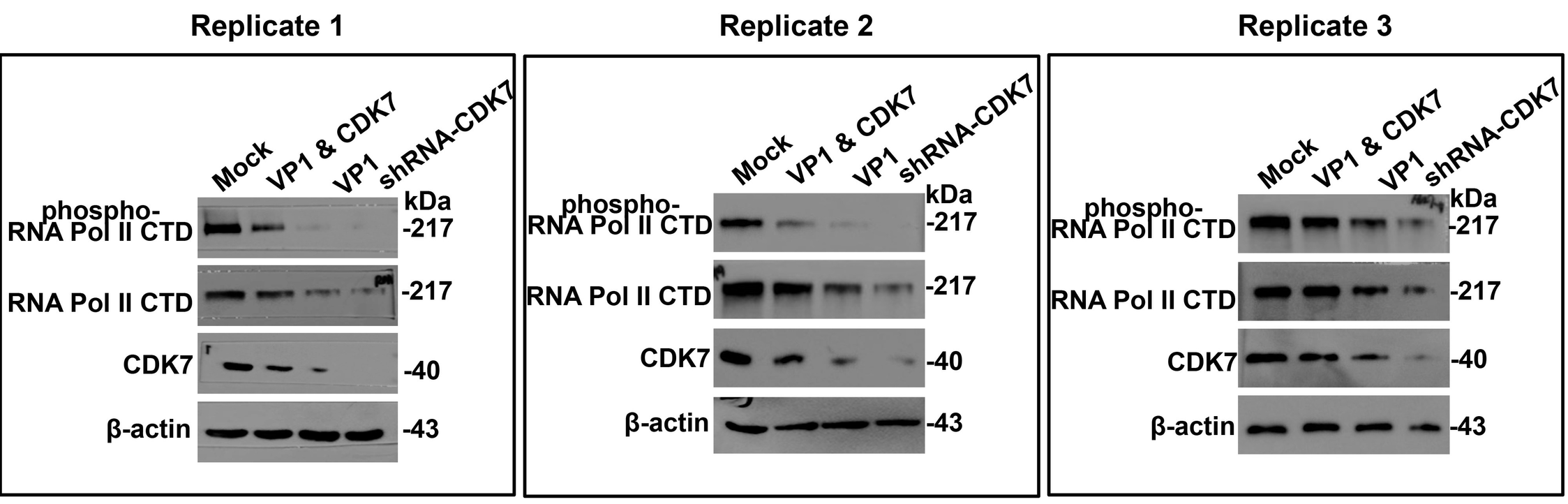

Supplement: S25 Fig — This result shows the raw data of the experiment repeated three times by independent researchers. Related to Fig 5D. (TIF) [file ppat.1008992.s025.tif]

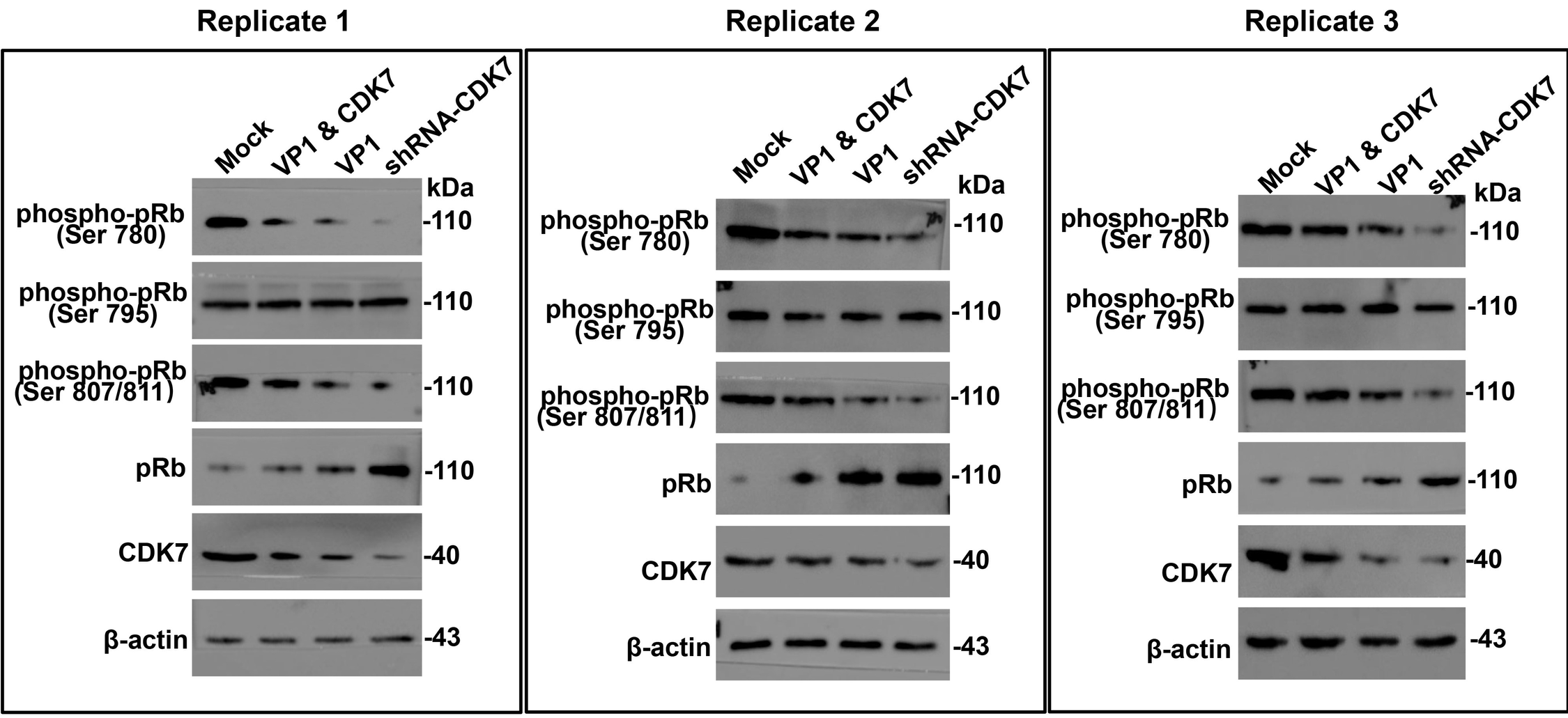

Supplement: S26 Fig — This result shows the raw data of the experiment repeated three times by independent researchers. Related to Fig 5E. (TIF) [file ppat.1008992.s026.tif]

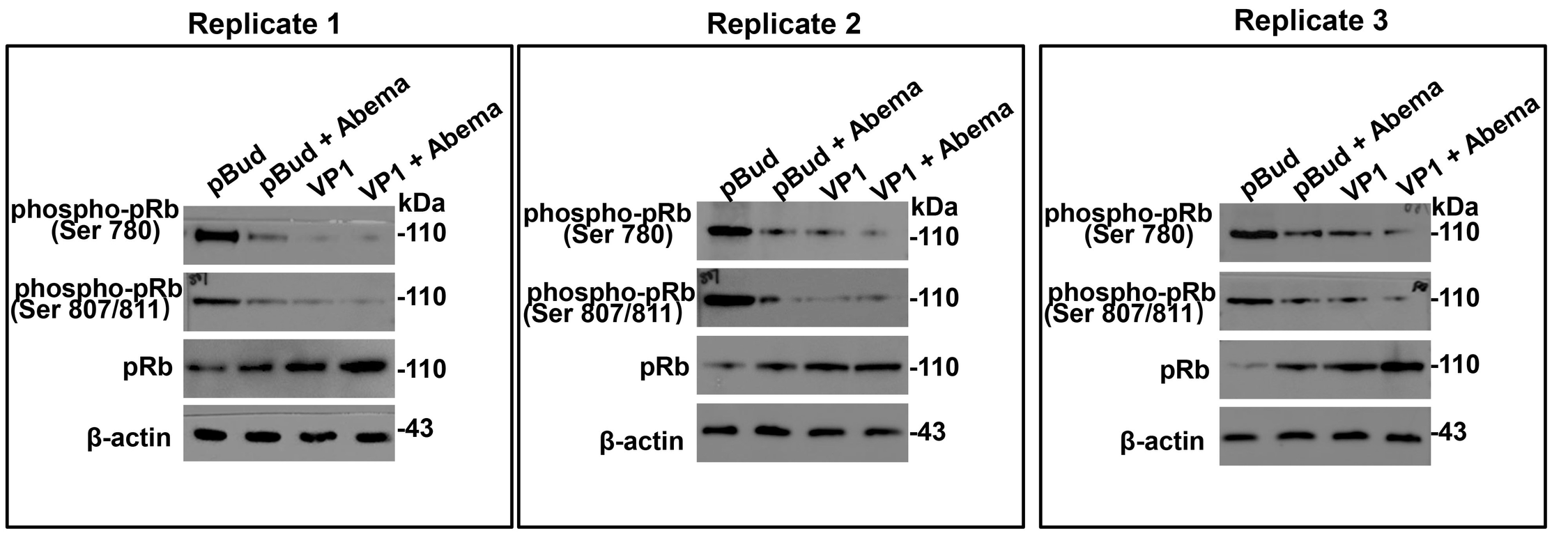

Supplement: S27 Fig — Western blotting analysis of the expression of phosphorylated/nonphosphorylated pRb with phosphor-pRb Ser 780, 807/811 antibodies. This result shows the raw data of the experiment repeated three times by independent researchers. Related to Fig 6C. (TIF) [file ppat.1008992.s027.tif]

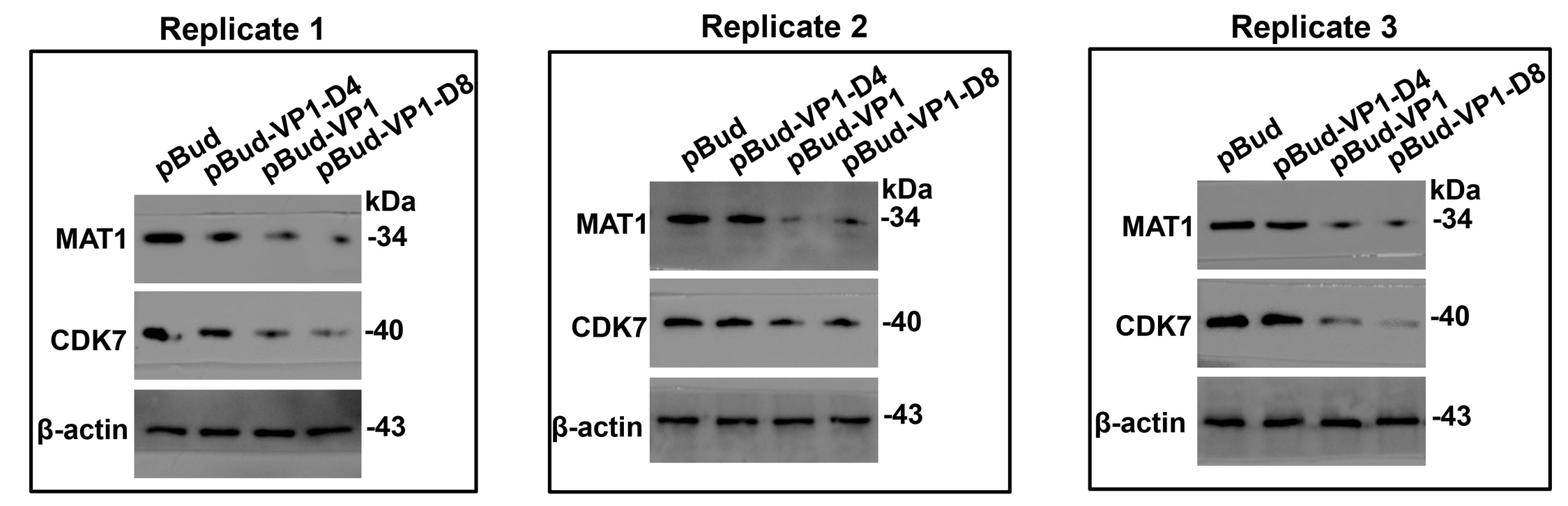

Supplement: S28 Fig — Immunoblot analysis of the abundance of MAT1 in pBud, VP1, VP1-D4 and VP1-D8 transfected cells. This result shows the raw data of the experiment repeated three times by independent researchers. Related to Fig 7C. (TIF) [file ppat.1008992.s028.tif]

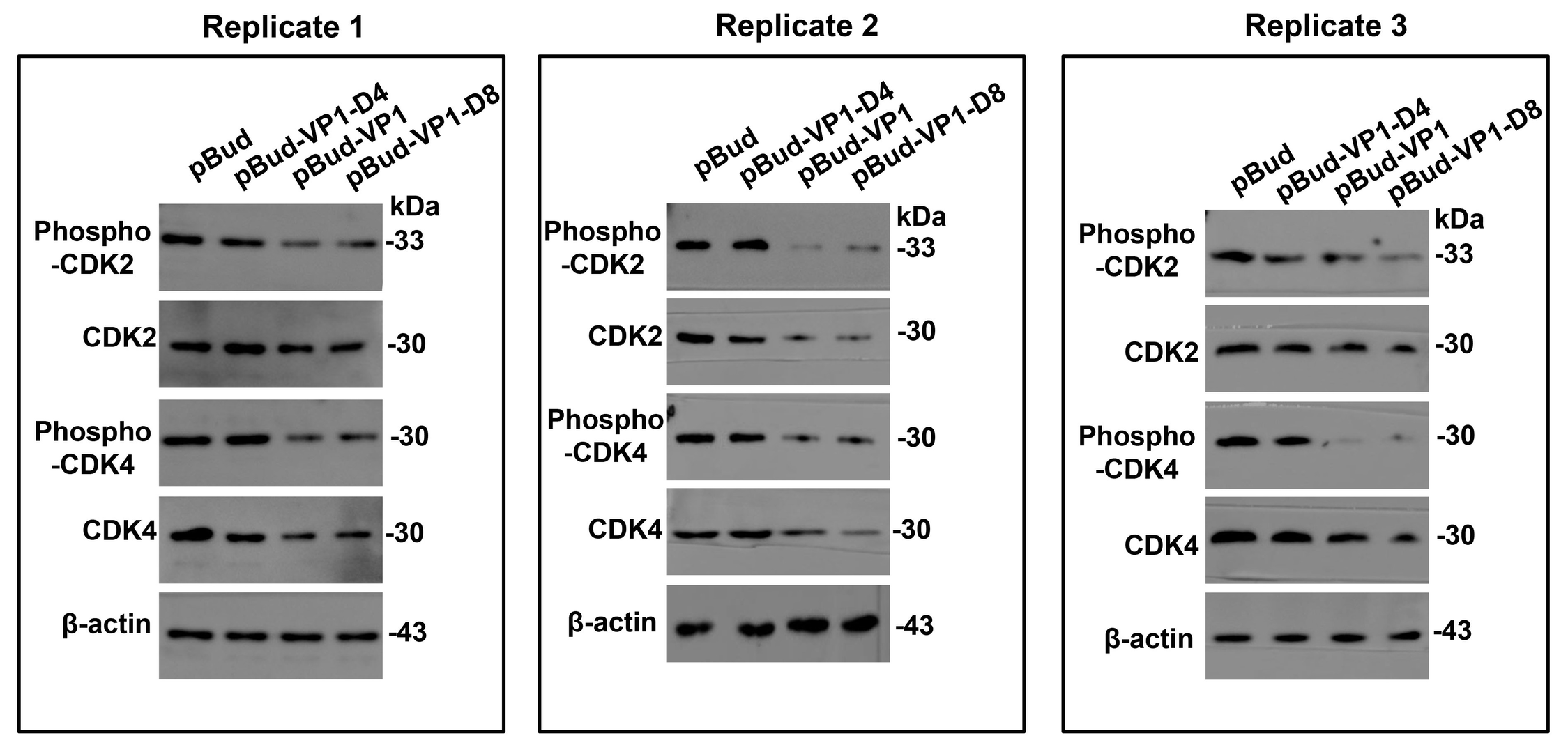

Supplement: S29 Fig — Western blot analysis of the accumulation of phosphorylated/nonphosphorylated CDK2 and CDK4 in different groups. This result shows the raw data of the experiment repeated three times by independent researchers. Related to Fig 7D. (TIF) [file ppat.1008992.s029.tif]

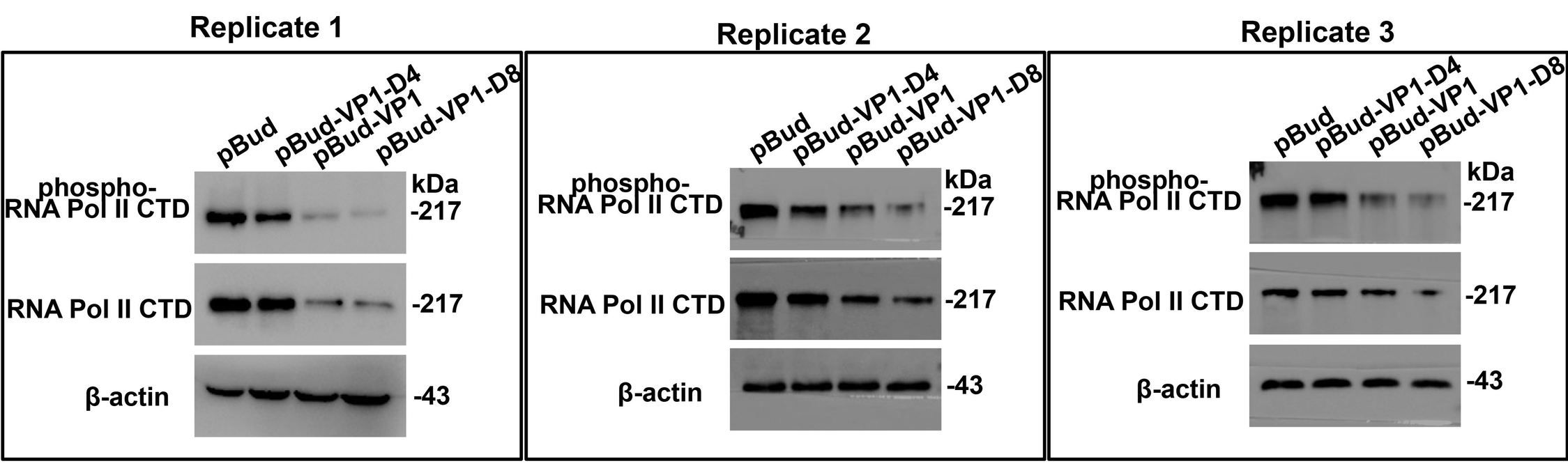

Supplement: S30 Fig — This result shows the raw data of the experiment repeated three times by independent researchers. Related to Fig 7E. (TIF) [file ppat.1008992.s030.tif]

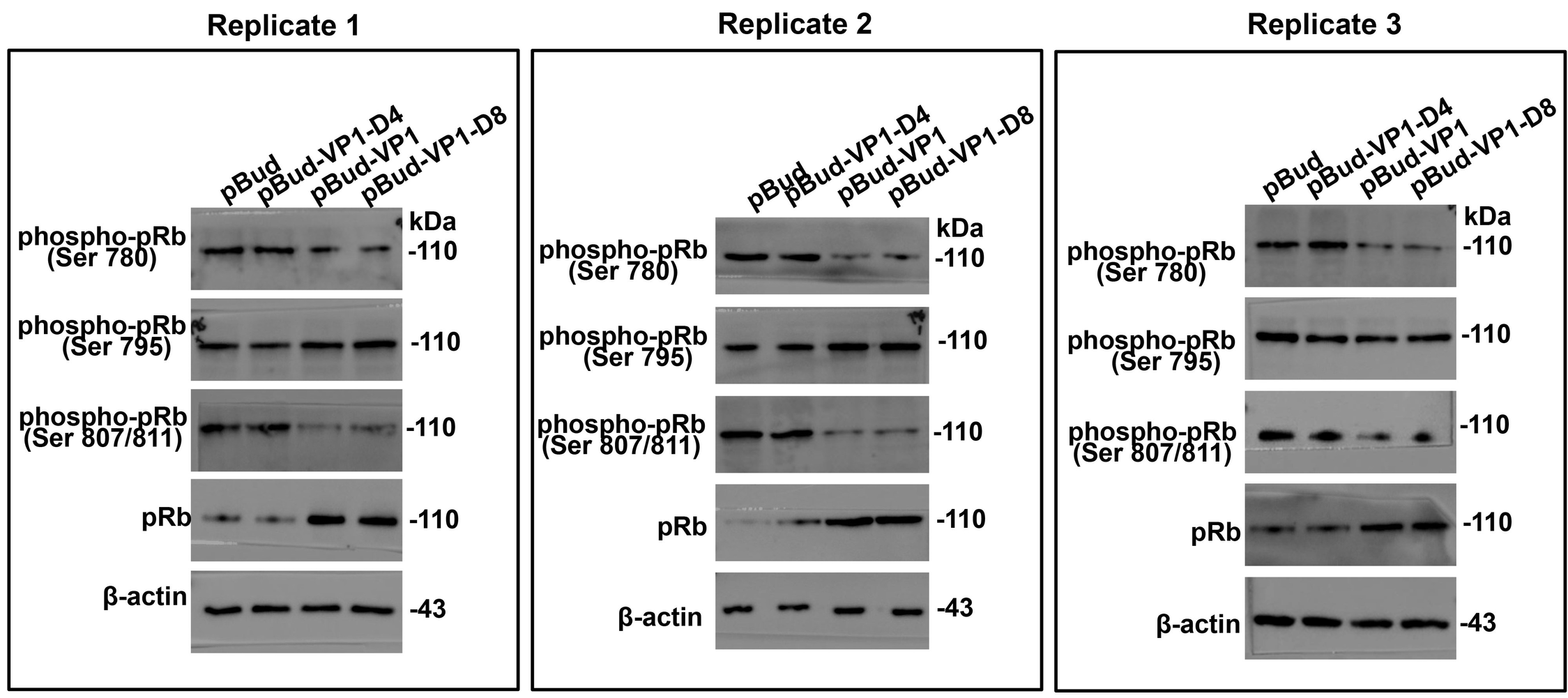

Supplement: S31 Fig — This result shows the raw data of the experiment repeated three times by independent researchers. Related to Fig 7F. (TIF) [file ppat.1008992.s031.tif]

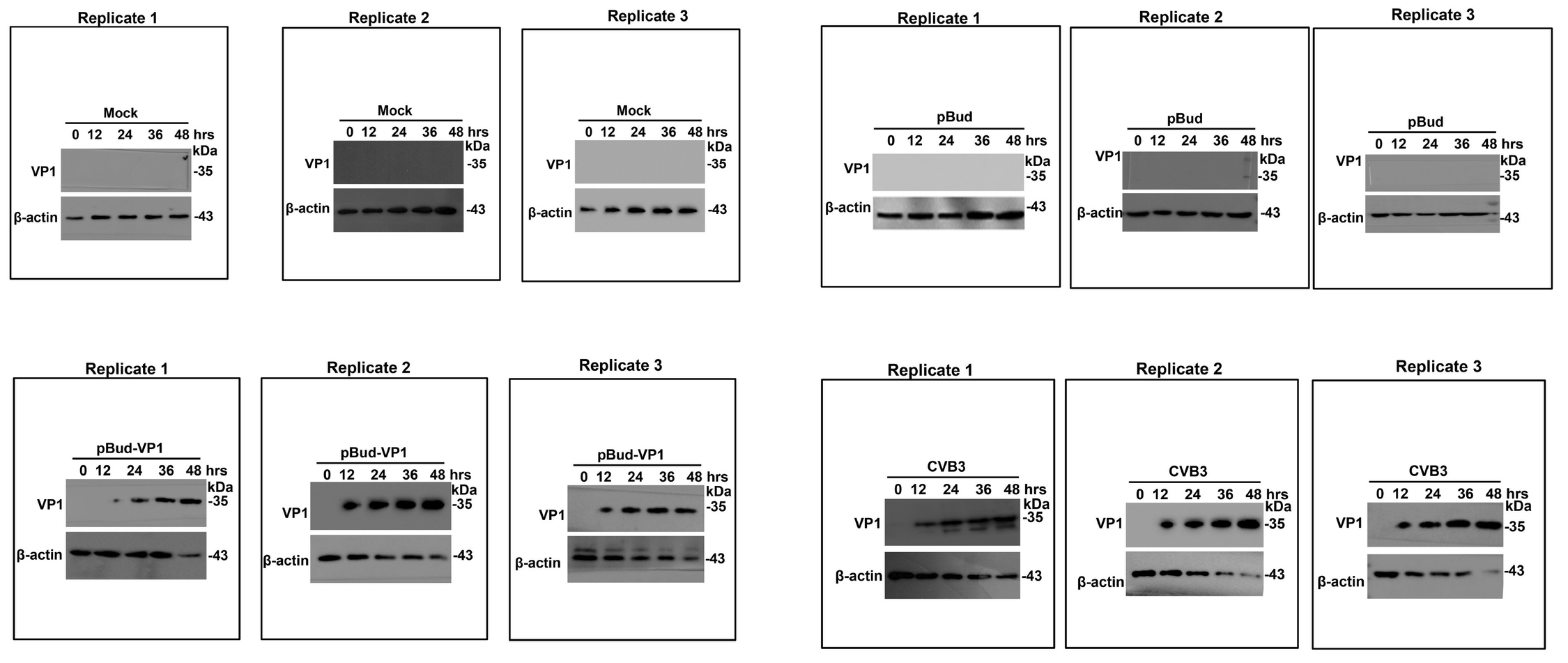

Supplement: S32 Fig — Western blot analysis of VP1 expression in Mock, pBud, pBud-VP1 and CVB3 groups. This result shows the raw data of the experiment repeated three times by independent researchers. Related to S2 Fig. (TIF) [file ppat.1008992.s032.tif]

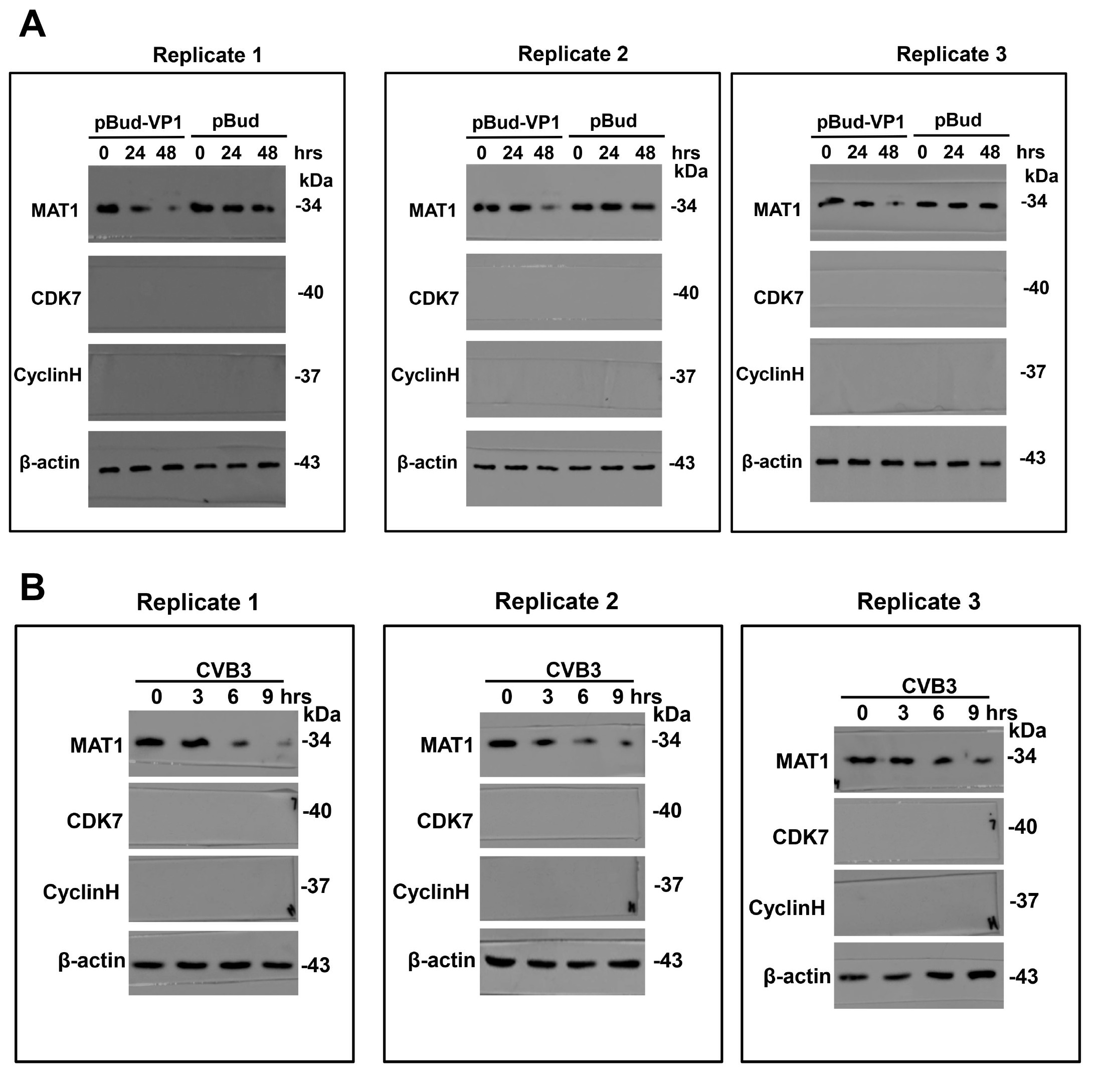

Supplement: S33 Fig — This result shows the raw data of the experiment repeated three times by independent researchers. Related to S10 Fig. (TIF) [file ppat.1008992.s033.tif]
