# Supplementary material for: CVB3 VP1 interacts with MAT1 to inhibit cell proliferation by interfering with Cdk-activating kinase complex activity in CVB3-induced acute pancreatitis
Source: PLoS Pathog. 2021 Feb 8;17(2):e1008992. doi: 10.1371/journal.ppat.1008992 (PMC7895353; doi:10.1371/journal.ppat.1008992)
Supplement: S1 Table — (DOCX) [file ppat.1008992.s034.docx]

**S1 Table. Primers used in plasmid construction in this study**

| Genes | Forward primer | Reverse primer |
| --- | --- | --- |
| pGBKT7-VP1 | GAATTCCATATGGTGGAAGACGCGATAA | AACTGCAGCCCTGATTGTTGTCCAAAT |
| pGADT7-MAT1 | CGGAATTCATGGACGATCAGGGTTGCC  R: | CGGGATCCTTAACTGGGCTGCCAGAAAAG |
| pBud-VP1 | GGAATTCCATATGGTGGAAGACGCGATAA | AACTGCAGCCCTGATTGTTGTCCAAAT |
| pBud-VP1-D2 | GGAATTCCATATGGTGGAAGACGCGATAA | AACTGCAGTCAGGACCTTGAATGGTAG |
| pBud-VP1-D3 | GGAATTCCATATGGTGGAAGACGCGATAA | AACTGCAGTCACTGTTGAGTACTTGTTA |
| pBud-VP1-D4 | GGAATTCCATATGGTGGAAGACGCGATAA | AACTGCAGTCACACGTATGAATCAACTTT |
| pBud-VP1-D5 | GGAATTCCATATGGTGGAAGACGCGATAA | AACTGCAGACCATCCGTCATAGAAATTTGA |
| pBud-VP1-D6 | GGAATTCCATATGGTGGAAGACGCGATAA | AACTGCAGATTGCGCCCGTATTTGTCATT |
| pBud-VP1-D7 | GGAATTCCATATGCATGTCAAAGCGTGG | AACTGCAGCTACCCTGATTGTTGTCCAAAT |
| pBud-VP1-D8 | GGAATTCCATATGGAATTTTCCAGGAACG | AACTGCAGCTACCCTGATTGTTGTCCAAAT |
| pBud-VP1-D9 | GGAATTCCATATGTGGCAAACATCTACG | AACTGCAGCTACCCTGATTGTTGTCCAAAT |
| pBud-VP1-D10 | GGAATTCCATATGCCCTCAACCACACAG | AACTGCAGCTACCCTGATTGTTGTCCAAAT |
| pBud-VP1-D11 | GGAATTCCATATGGAGTCAACCATAGAG | AACTGCAGCTACCCTGATTGTTGTCCAAAT |
| pBud-VP1-D12 | GGAATTCCATATGTGGCAAACATCTACG | AACTGCAGACCATCCGTCATAGAAATTTGA |
| pBud-VP1-D13 | GGAATTCCATATGCCCTCAACCACACAG | AACTGCAGACCATCCGTCATAGAAATTTGA |
| pBud-VP1-D14 | GGAATTCCATATGGAGTCAACCATAGAG | AACTGCAGACCATCCGTCATAGAAATTTGA |
| pBud-VP1-D4 | CCGCTCGAGATGGAAGACGCGATAACAG | GAAGATCTTGCCACACGTATGAATCAACTTT |
| pBud-VP1-D8 | CCGCTCGAGATGTCTGAATTTTCCAGGA | GAAGATCTGATTGTTGTCCAAATGCGCCC |
| pCAG-Flag-CDK7 | CAAGGATGACGATGACAAGGGTACCATGGCTCTGGACGTG | GGGTATGATTTCCCGGTACCTTTTTTTTTTTTTTTTTTCAG |
| pAdtrack-Flag-VP2 | TCTGGTACCGTCGACGCGGCCGCATGGCTAGCTGGAGCCAC | GGTGGATCGGATATCACTAAGCTTAGGCGTCGGCAAATCCCAG |
| pAdtrack-Flag-VP3 | GATGACGATGACAAGGGTACCGGCTTACCAACCATGAATAC | GGGTATGATTTCCCGGTACCCTGGAAAAAGTTTTGCTGC |
| pAdtrack-Flag-VP4 | GATGACGATGACAAGGGTACCATGGGAGCTCAAGTATC | CAACCCATACTAAAGGGCCATGGCAACTCGACCATCAC |
| TNFα | CCTGCCCCAATCCCTTTATT | TTGAACAGCCTCACAGAGCAGA |
| IL-4 | TTGAACAGCCTCACAGAGCAGA | GTTGTGTTCTTGGAGGCAGCA |
| IL-6 | TGCGTCCGTAGTTTCCTTCT | GCCTCAGACATCTCCAGTCC |
| IL-10 | AATAAGGTTTCTCAAGGGGCT | AGAACCAAGACCCAGACATCAA |
| GAPDH | GAAGGTGGTGAAGCAGGCATC | GTGGGAGTTGCTGTTGAAGTC |
